# Ultrasound‐Responsive Piezoelectric Fibrous Membrane Promotes Neurological Recovery After TBI by Modulating Microglial Polarization via Restoring Mitochondrial Dynamic Homeostasis

**DOI:** 10.1002/advs.202600081

**Published:** 2026-09-21

**Authors:** Wei Li, Runzhe Huang, Qingyuan Wu, Pengbo Zhou, Dangli Ren, Jingjing Wang, Hanjie Niu, Zemeng Li, Hongtao Sun, Huiyu Liu

**Affiliations:** ^1^ The First School of Clinical Medical Lanzhou University Lanzhou Gansu People's Republic of China; ^2^ Center Laboratory for Neurological Diseases Characteristic Medical Center of People's Armed Police Forces Tianjin People's Republic of China; ^3^ Beijing Advanced Innovation Center for Soft Matter Science and Engineering State Key Laboratory of Organic‐Inorganic Composites Bionanomaterials & Translational Engineering Laboratory Beijing Key Laboratory of Green Chemicals Biomanufacturing Beijing Laboratory of Biomedical Materials Beijing University of Chemical Technology Beijing People's Republic of China; ^4^ Department of Chemistry Key Laboratory of Bioorganic Phosphorus Chemistry & Chemical Biology Tsinghua University Beijing People's Republic of China

**Keywords:** LIPUS, microglial polarization, mitochondrial dysfunction, neural repair, piezoelectric fibrous membrane, TBI

## Abstract

Mitochondrial dysfunction is a core pathological mechanism underlying secondary injury following traumatic brain injury, with the resulting oxidative stress and inflammatory cascade being key contributors to neurological deficits and poor clinical outcomes. This study developed an ultrasound‐responsive piezoelectric fibrous membrane that, under low‐intensity pulsed ultrasound (LIPUS) stimulation, generates controllable piezoelectric signals directly at the injury site to precisely regulate mitochondrial function in microglia. This regulation effectively restores mitochondrial dynamics homeostasis and enhances mitochondrial membrane potential (ΔΨm) stability, significantly suppressing the abnormal generation of mitochondrial superoxide and cellular reactive oxygen species (ROS). It promotes the polarization of microglia toward a neuroprotective M2 phenotype, reduces the expression of pro‐inflammatory cytokines TNF‐α, IL‐1β, and IL‐6, and enhances the activity of the antioxidant enzyme superoxide dismutase (SOD). In a TBI animal model, this therapeutic strategy markedly alleviated pathological brain damage, improved neuronal survival, ameliorated neurological deficits and spatial memory impairment, and facilitated the polarization of microglia toward an anti‐inflammatory (M2) phenotype in the injured area. This study provides a novel strategy for TBI treatment by targeting mitochondrial function regulation, offering potential to overcome the therapeutic challenges in neuroimmunometabolic modulation.

## Graphical abstract

We demonstrate a novel therapy for traumatic brain injury TBI using a LIPUS‐responsive Piezoelectric fibrous membrane. It targets microglial mitochondria via in situ electrical signals, restoring homeostasis and reducing oxidative stress to promote an anti‐inflammatory M2 phenotype. This approach significantly improves neuronal survival, functional recovery, and memory in animal models, providing a new strategy for TBI treatment.

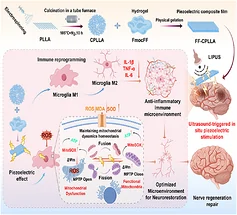

## 1 Introduction

Traumatic brain injury (TBI) represents a significant cause of global disability and mortality in young adults. According to a report by the World Health Organization [1], approximately 50 million people suffer from TBI globally each year, with more than half of the patients experiencing long‐term cognitive and motor impairments, placing a heavy burden on healthcare systems. Current clinical treatments primarily focus on hematoma evacuation and intracranial pressure control during the acute phase. However, effective interventions for secondary brain injury‐particularly the neuroinflammatory cascade and oxidative stress damage‐remain lacking, often resulting in poor neurological recovery [2].

A growing body of recent research has demonstrated that mitochondrial dysfunction in microglia is a central mechanism underlying secondary neural injury following TBI [3, 4]. After TBI, microglial mitochondria undergo a dynamical imbalance and collapse of the mitochondrial membrane potential (ΔΨm), leading to electron transport chain (ETC) uncoupling and triggering a burst of reactive oxygen species (ROS). Excessive ROS directly oxidizes mitochondrial DNA (mtDNA) and lipid membranes, further exacerbating mitochondrial damage and forming a vicious cycle [5, 6]. MtDNA leaked into the cytoplasm can activate the cGAS–STING pathway, driving NF‐κB phosphorylation and subsequently promoting the expression of pro‐inflammatory factors such as TNF‐α, IL‐1β, and IL‐6 [7]. Concurrently, mitochondrial calcium overload (Ca^2+^overload) activates the NLRP3 inflammasome, further amplifying the inflammatory cascade [8], collectively aggravating neural injury. Additionally, the modulation of microglial polarization states has emerged as a crucial strategy for treating neuroinflammatory diseases. Zhang et al. [9]. developed calcium‐retinoic acid nanoparticles that effectively promote the polarization of microglia toward the anti‐inflammatory M2 phenotype. This shift indirectly facilitates the differentiation of neural stem cells into neurons, presenting a promising therapeutic avenue for neuroinflammatory disorders. Moreover, studies have confirmed [10] that the aforementioned mitochondrial dysfunction and associated inflammatory responses directly inhibit the polarization of microglia toward the neuroprotective M2 phenotype—characterized by the release of IL‐10 and TGF‐β—while promoting their polarization toward the pro‐inflammatory M1 phenotype, ultimately resulting in neuronal apoptosis and impaired synaptic plasticity. Therefore, targeted restoration of mitochondrial function is considered a critical breakthrough for reversing the neuroinflammatory microenvironment [11].

Electrical stimulation (ES) therapy has been demonstrated to enhance ATP synthesis, mitigate oxidative damage, and promote neuronal regeneration through the modulation of ion channels and mitochondrial metabolic pathways [12, 13, 14]. A study by Lu et al. [15] indicated that ES effectively facilitates cell migration, adhesion, and axonal growth (e.g., Schwann cell activation and myelination) during peripheral nerve regeneration, significantly improving functional recovery. These findings suggest that ES may also hold potential for central nervous system repair. However, the clinical translation of this ES approach is hampered by several challenges: the reliance on complex circuitry and external power sources, the risk of inflammatory responses from implanted electrodes, and the necessity of a secondary surgery for their removal [16, 17]. Piezoelectric materials have demonstrated unique advantages in neural interface engineering due to their ability to convert mechanical energy into electrical signals [18]. Studies have shown that piezoelectric scaffolds can provide an interface that promotes the adhesion, proliferation, and angiogenesis of Schwann cells in vitro; meanwhile, mechanoelectrical stimulation generated in vivo can establish a biomimetic conductive microenvironment that enhances nerve conduction, facilitates axonal myelination, and improves motor function recovery [19]. Although extensive studies have focused on piezoelectric materials for tissue regeneration, particularly peripheral nerve repair [20, 21, 22], their potential for treating brain trauma remains underexplored. This includes their role in modulating the neuroimmune microenvironment by ameliorating mitochondrial dysfunction [23].

Based on this, the present study successfully developed a novel in situ ultrasound‐responsive piezoelectric fibrous membrane (FF–CPLLA). This material was fabricated by subjecting a poly(L‐lactic acid) (PLLA) substrate to controlled calcination, resulting in a crystalline PLLA (CPLLA) fibrous membrane with higher crystallinity and significantly enhanced piezoelectric properties. Through molecular self‐assembly technology, Fmoc‐diphenylalanine (FmocFF) was introduced onto the film surface. FmocFF molecules spontaneously assembled into a nanofibrous network under mild conditions, forming a dense coating on the CPLLA, which further improved the overall biocompatibility and cell adhesion of the material. Under low‐intensity pulsed ultrasound (LIPUS) stimulation, the piezoelectric effect of the material is activated, enabling targeted action in the injured brain region. It modulates mitochondrial function and polarization phenotypes of microglia, thereby regulating the neuroimmune microenvironment and ultimately promoting brain tissue repair and functional recovery. The research scheme is illustrated in Figure 1.

**FIGURE 1:**
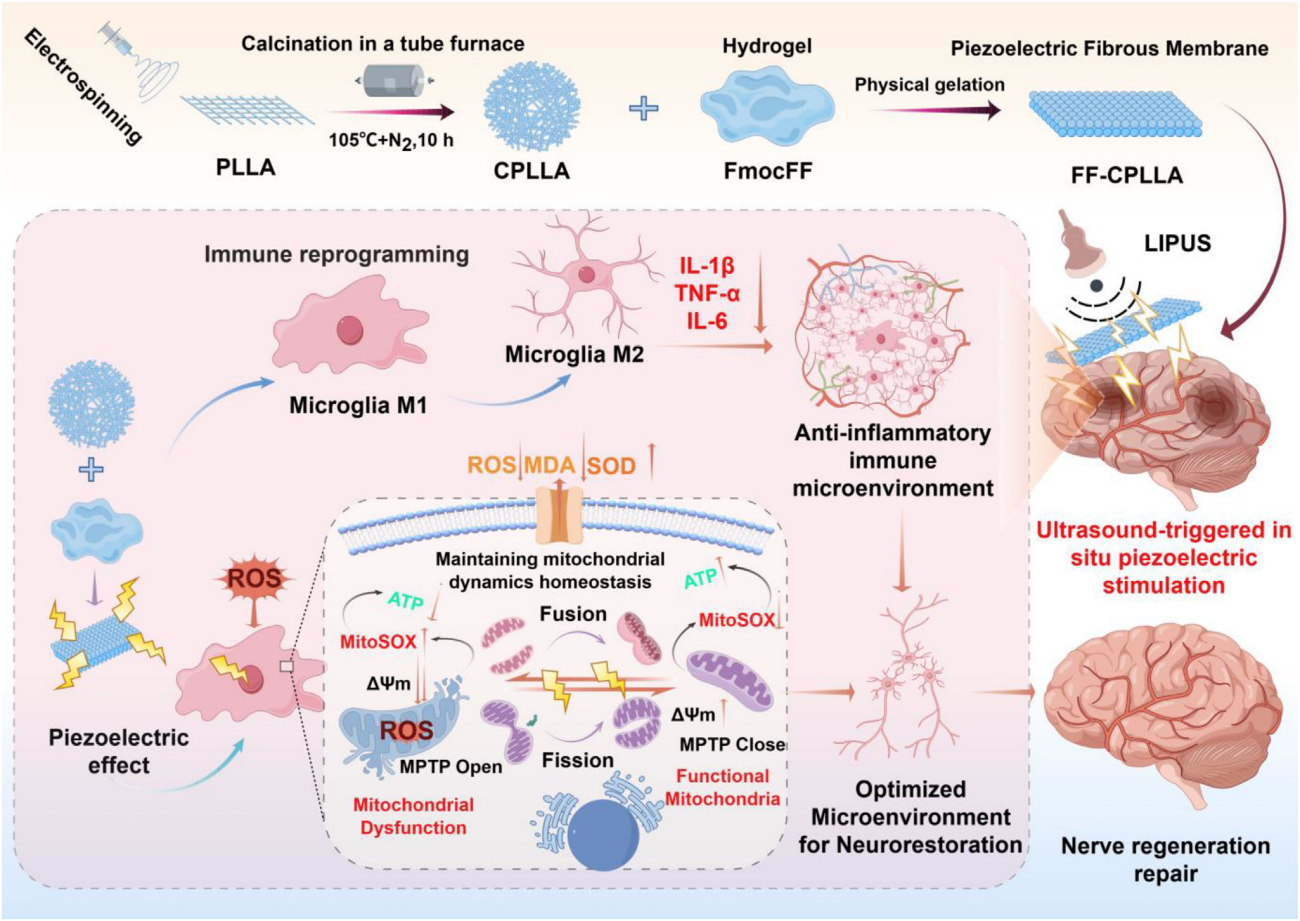
A piezoelectric fibrous membrane system designed for the treatment following traumatic brain injury (TBI). This system utilizes low‐intensity pulsed ultrasound (LIPUS) to drive the in situ generation of controllable piezoelectric signals at the injury site. This process aims to precisely modulate mitochondrial function and the polarization phenotype of microglia, thereby ameliorating the neuroimmune microenvironment. The ultimate therapeutic goals are the attenuation of histopathological damage, enhancement of neuronal survival, and the promotion of neural repair.

## 2 Results and Discussion

### 2.1 Synthesis and Characterization of Piezoelectric Fibrous Membrane

In this study, PLLA fibrous membranes were initially fabricated via electrospinning. The obtained PLLA fibrous membranes were subsequently subjected to annealing under nitrogen atmosphere to produce crystallized PLLA (CPLLA) fibrous membranes. Thereafter, FmocFF was dissolved in dimethyl sulfoxide (DMSO) and mixed with an aqueous sodium chloride (NaCl) solution. The mixture was evenly drop‐cast onto the surface of the CPLLA fibrous membranes and allowed to stand at room temperature to induce hydrogel formation. The FF–CPLLA composite fibrous membranes were finally obtained after freeze‐drying (Figure 2A).

**FIGURE 2:**
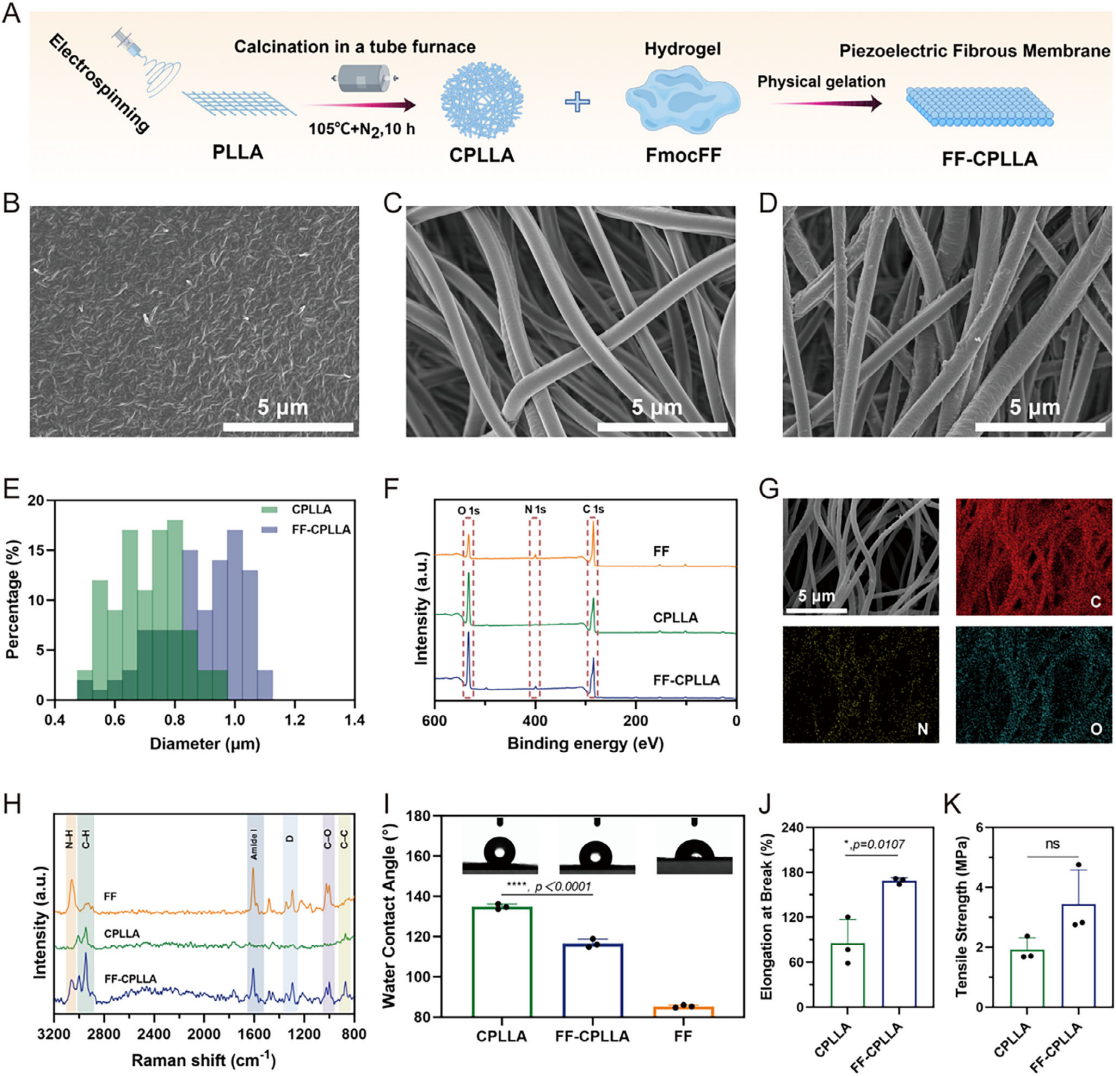
Synthesis and characterization of the FF‐CPLLA piezoelectric fibrous membrane. (A) Schematic illustration of the fabrication process of the FF‐CPLLA piezoelectric fibrous membrane. (B) Scanning electron microscopy (SEM) image of the FF hydrogel. Scale bar, 5 µm. (C) SEM image of the CPLLA fibrous membranes. Scale bar, 5 µm. (D) SEM image of the FF‐CPLLA composite fibrous membranes. Scale bar, 5 µm. (E) Diameter distribution of CPLLA and FF‐CPLLA fibrous membranes. (F) X‐ray photoelectron spectroscopy (XPS) spectra of the FF hydrogel, CPLLA fibrous membranes, and FF‐CPLLA fibrous membranes. (G) Elemental mapping images of C, N, and O for the FF‐CPLLA fibrous membranes. Scale bar, 5 µm. (H) Raman spectra of the FF hydrogel, CPLLA fibrous membranes, and FF‐CPLLA fibrous membranes. (I) Contact angle measurements of the FF hydrogel, CPLLA fibrous membranes, and FF‐CPLLA fibrous membranes. Data are presented as mean ± SD; *n* = 3. One‐way ANOVA was used for statistical analysis. (J) Elongation at break of CPLLA and FF‐CPLLA fibrous membranes. Data are presented as mean ± SD; *n* = 3. One‐way ANOVA was used for statistical analysis. (K) Tensile strength of CPLLA and FF‐CPLLA fibrous membranes. Data are presented as mean ± SD; *n* = 3. One‐way ANOVA was used for statistical analysis.

Scanning electron microscopy (SEM) results revealed that the pure FF hydrogel exhibited a dense fibrous network structure (Figure 2B), while the CPLLA fibrous membranes showed a relatively uniform fibrous morphology (Figure 2C). In contrast, the FF–CPLLA composite fibrous membranes combined features of both, displaying a rougher fiber surface (Figure 2D), indicating successful self‐assembly of the FF hydrogel on the CPLLA fibrous membranes. Further diameter distribution analysis (Figure 2E) demonstrated that the average fiber diameter of the CPLLA fibrous membranes was 0.7079 ± 0.1095 µm, whereas that of the FF–CPLLA composite fibrous membranes increased to 0.8843 ± 0.1401 µm. Concurrently, analysis of the fibrous network revealed that the average fiber spacing decreased from approximately 2.3652 ±1.4098 µm in CPLLA to 1.8122 ± 1.2776 µm in FF‐CPLLA (Figure S1A), while the porosity was measured at 50.45 ± 3.48% and 43.84 ± 4.28% (Figure S1B), respectively. These results suggest that the incorporation of FF promoted fiber coarsening, leading to a more stable fibrous structure in the composite fibrous membranes. X‐ray photoelectron spectroscopy (XPS) analysis (Figure 2F) revealed distinct nitrogen signals in the FF–CPLLA composite fibrous membranes. Compared with pure FF hydrogel and pure CPLLA, the composite fibrous membranes exhibited characteristic peaks from both components, along with slight chemical shifts in the C═O, C─O, and CO–NH peaks (Figure S2A–F). The content of –NH_3_^+^ increased from 19.16% in the pure FF hydrogel to 26.54% in the FF–CPLLA composite. These results collectively indicate the successful modification of the CPLLA surface with the FF hydrogel, likely mediated through hydrogen bonding or *π*–*π* stacking interactions. Elemental mapping (Figure 2G and Figure S3A,B) further confirmed the uniform distribution of C, N, and O elements throughout the composite fibrous membranes. The nitrogen content increased from 0.82% in CPLLA to 1.80% in FF–CPLLA, demonstrating effective loading of FF on the material surface. Raman spectroscopy (Figure 2H) showed that the FF–CPLLA composite retained characteristic peaks of both FF and CPLLA, including the Amide I band of FF and the C–H vibration of PLLA, indicating that the structural features of both components were preserved during the composite formation. The degradation behavior of FF‐CPLLA fibrous membranes was evaluated in simulated cerebrospinal fluid (CSF). SEM images revealed a gradual surface erosion and fiber thinning over 1–4 weeks (Figure S4), accompanied by a time‐dependent increase in mass loss (Figure S5A) and lactic acid release (Figure S5B), indicating a controlled and progressive degradation process under CSF‐mimicking conditions.

Contact angle measurements (Figure 2I) showed that the pure CPLLA fibrous membranes exhibited a large contact angle, indicating strong hydrophobicity, while the pure FF hydrogel displayed a small contact angle, demonstrating hydrophilic properties. The contact angle of the FF–CPLLA composite fibrous membranes fell between those of the two individual components. This result suggests that the incorporation of FF improved the wettability of the CPLLA surface, endowing it with moderate hydrophilicity and stability, which is beneficial for subsequent applications in biological environments. The mechanical properties of both CPLLA and FF–CPLLA were also evaluated. Stress–strain curves (Figure S6) revealed typical nonlinear elastic behavior in both samples. Force–displacement curves (Figure S7) indicated that FF–CPLLA generally exhibited higher force values than CPLLA at the same displacement, suggesting an enhancement in the overall stiffness of the fibrous structure after the incorporation of the FF hydrogel. The calculated elongation at break was significantly higher for FF–CPLLA (167%) compared to CPLLA (85%), indicating a notable improvement in the ductility and toughness of the composite material (Figure 2J). Although the tensile strength (Figure 2K) and Young's modulus (Figure S8) of FF–CPLLA showed an increasing trend compared to CPLLA, the differences were not statistically significant. These findings suggest that the composite structure primarily exhibits enhanced ductility. This behavior may originate from non‐covalent interactions between FF and CPLLA. These reversible interactions can continuously break and reform during stretching, providing additional energy dissipation pathways. Meanwhile, the molecular‐level interactions between FF and CPLLA chains may facilitate the formation of an interpenetrating network‐like structure, thereby contributing to the improved overall mechanical performance.

Poly(L‐lactic acid) (PLLA), a semi‐crystalline polymer characterized by C═O dipoles, exhibits a non‐polar nature because these dipoles are randomly aligned in the main chain under ambient conditions, rendering the material non‐polar [24]. After calcination treatment, the CPLLA fibrous membranes exhibited a distinct diffraction peak at approximately 16.4° (Figure S9), indicating a significant increase in crystallinity. Higher crystallinity generally implies a more ordered molecular chain alignment, which not only improves the structural stability of the material but also provides a better foundation for maintaining dipole orientation, thereby contributing to enhanced piezoelectric performance.

Atomic force microscopy (AFM) characterization revealed that the FF‐CPLLA fibrous membranes exhibit a uniform and continuous fibrous architecture (Figure 3A). The nanoscale piezoelectric behavior was subsequently investigated by piezoresponse force microscopy (PFM). The PFM phase images displayed pronounced light‐dark contrast across different regions of the FF‐CPLLA fibrous membranes, indicating heterogeneous polarization orientations within the porous fibrous network (Figure 3B). Correspondingly, the PFM amplitude images exhibited strong and spatially heterogeneous piezoresponse signals, with variations in response intensity associated with local microstructural differences, suggesting anisotropic piezoelectric behavior on the fibrous membrane surface (Figure 3C). Local piezoresponse spectroscopy further confirmed the robust piezoelectric activity of the FF‐CPLLA fibrous membranes. As shown in Figure 3D, a typical butterfly‐shaped amplitude curve accompanied by clear phase switching behavior was observed, demonstrating stable and reversible polarization under an applied electric field. Notably, the piezoelectric responses were determined by PFM‐based local measurements at the single‐fiber or nanoscale domain level, with d33 values extracted from the slope of the amplitude‐bias curves. Based on the same PFM methodology, the piezoelectric properties of PLLA, CPLLA, and FF hydrogel were systematically evaluated for comparison (Figures S10 and S11). For PLLA and CPLLA fibrous membranes, the PFM phase images revealed distinct polarization contrasts, while the corresponding amplitude images exhibited identifiable piezoelectric domains, indicating randomly distributed polarization regions within the fibrous membranes. Quantitative analysis showed that the piezoelectric coefficient (d33) of pristine PLLA fibrous membranes was 8.32 ± 0.88 pm/V, which increased significantly to 27.24 ± 2.04 pm/V after calcination to form CPLLA fibrous membranes, demonstrating an effective enhancement of piezoelectric performance. The FF hydrogel also exhibited an intrinsic piezoelectric response, with a d33 value of 26.73 ± 2.60 pm/V. Notably, the FF‐CPLLA fibrous membranes displayed the highest piezoelectric coefficient of 34.10 ± 5.48 pm/V, indicating that the incorporation of FF synergistically enhanced the piezoelectric performance of the composite fibrous membranes (Figure 3E).

**FIGURE 3:**
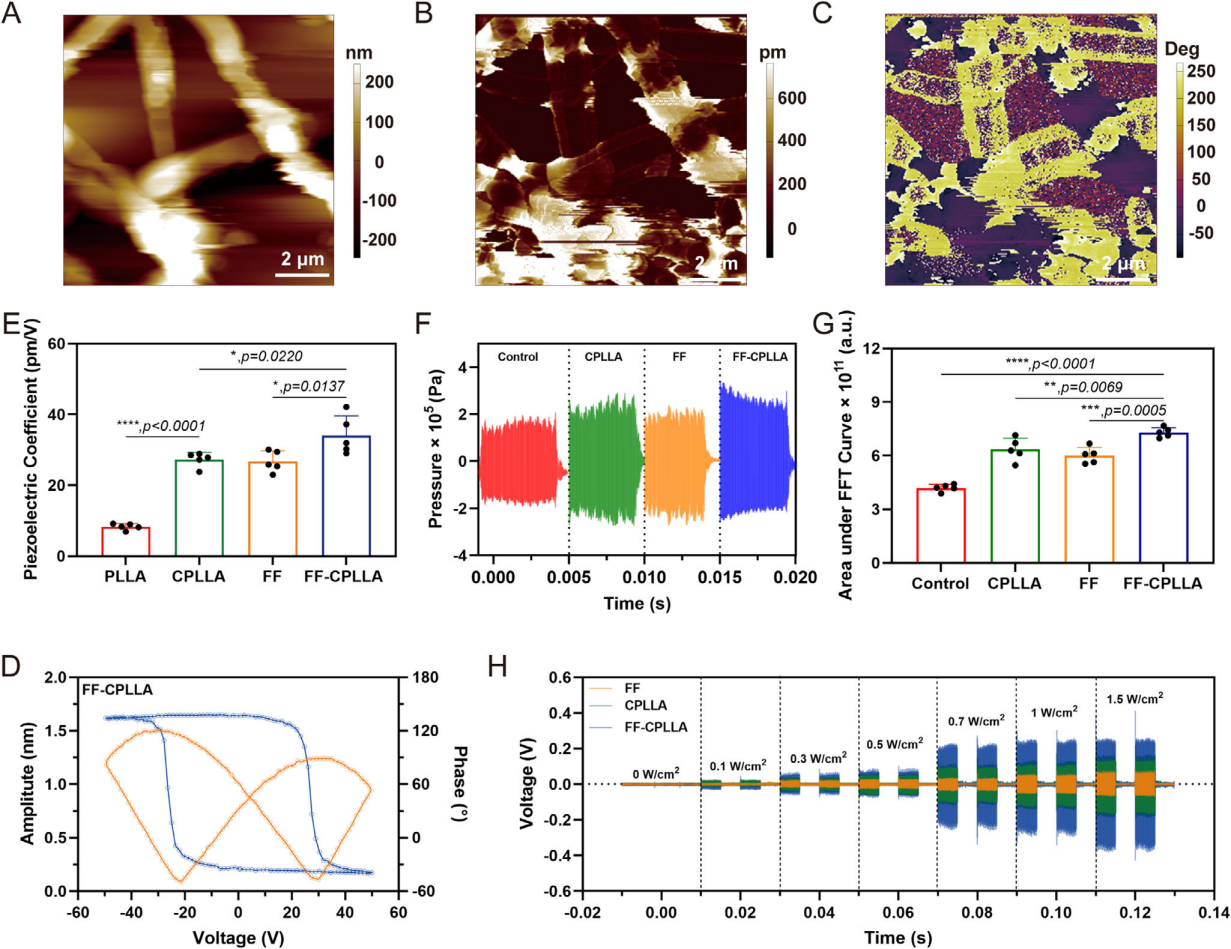
Actuation and Modulation of Piezoelectric Signals by Ultrasound: (A) Atomic force microscopy (AFM) image of FF‐CPLLA. Scale bar, 2 µm. (B) Piezoresponse force microscopy (PFM) amplitude image of FF‐CPLLA. Scale bar, 2 µm. (C) PFM phase image of FF‐CPLLA. Scale bar, 2 µm. (D) Amplitude‐bias and phase‐bias hysteresis curves of the FF‐CPLLA fibrous membranes. (E) Comparison of the piezoelectric coefficients of PLLA, CPLLA, FF, and FF‐CPLLA. Data are presented as mean ± SD; *n* = 5. One‐way ANOVA was used for statistical analysis. (F) Acoustic pressure signals generated by different groups in response to ultrasound stimulation. (G) The integration of the FFT spectrum. Data are presented as mean ± SD; *n* = 5. One‐way ANOVA was used for statistical analysis. (H) Voltage output of the FF hydrogel, CPLLA fibrous membranes, and FF‐CPLLA fibrous membranes under ultrasound irradiation at different power levels.

To further evaluate the acoustic response of different systems under ultrasound (US) excitation, a hydrophone was employed to monitor the sound pressure signals generated by the Control, CPLLA, FF, and FF‐CPLLA groups under identical US conditions (Figure 3F). From the sound pressure–time profiles, the FF‐CPLLA group exhibited markedly higher instantaneous sound pressure amplitudes compared to the other three groups, indicating a significantly enhanced acoustic response under US stimulation. The acquired signals were subsequently processed by Fast Fourier Transformation (FFT) to obtain the corresponding frequency‐domain spectra (Figure S12). All groups displayed a pronounced main peak centered at 1.0 MHz, which corresponds to the applied US frequency. To quantitatively compare cavitation intensity among different groups, the FFT spectra were numerically integrated over a frequency band around the ultrasound fundamental frequency [25]. The FF‐CPLLA group exhibited a significantly larger integrated spectral area than the other three groups, with statistically significant differences (Figure 3G). These results indicate that incorporation of FF modifies the acoustic response of the CPLLA fibrous membrane under ultrasound exposure and may contribute to its enhanced ultrasound responsiveness. Whether this altered acoustic response contributes to the biological effects through cavitation‐related mechanisms requires further dedicated investigation.

Corresponding voltage output results under ultrasound excitation (Figure 3H) further revealed that the FF‐CPLLA fibrous membranes generate significant electrical signals at different ultrasound power levels, with the output voltage gradually increasing as the ultrasound power rises, demonstrating its superior acoustic‐responsive piezoelectric characteristics. To further evaluate the practical transcranial applicability, the voltage outputs of FF‐CPLLA fibrous membranes were measured in air, cerebrospinal fluid (CSF), and through an ex vivo rat skull (Figures S13 and S14A). Although a moderate attenuation of the output voltage was observed in CSF, the FF‐CPLLA fibrous membranes still maintained a relatively high electrical output under transcranial ultrasound conditions, indicating efficient ultrasound penetration and piezoelectric signal generation through biological media (Figure S14B). In addition, temperature monitoring under both in vitro and in vivo ultrasound exposure showed negligible temperature changes (Figure S15), indicating that appreciable heating was not induced under the applied ultrasound conditions and reducing the likelihood that thermal effects were a major contributor to the observed biological responses.

The acoustic field at the sample location was mapped in two dimensions using a calibrated needle hydrophone. The peak‐pressure distributions in the X–Y and X–Z planes showed that acoustic energy was concentrated within the effective field encompassing the sample region (Figure S16A,B). The maximum peak pressure at the sample location was 35.6 kPa (Figure S16C), while the mean peak pressure within the sample region (the central 0.5 × 0.5 cm area in the X–Y plane) was 19.4 ± 6.4 kPa. The −6 dB lateral beam widths were 15.4 × 15.1 mm^2^, and the axial focal length was 9.3 mm, corresponding to an estimated focal volume of 1132.3 mm^3^, which covered the membrane used in this study (Table S1). Notably, the axial pressure profile did not show regular λ/2‐periodic modulation (approximately 0.74 mm at 1 MHz in water; Figure S16D), indicating that the acoustic field at the sample location was not dominated by pronounced standing‐wave artefacts. These measurements provide a quantitative basis for the actual pressure level, spatial distribution, and effective exposure range of the ultrasound stimulus used in the subsequent experiments.

In summary, the FF‐CPLLA composite fibrous membranes demonstrate a uniform morphological structure, enhanced mechanical properties, optimized hydrophilicity, and significantly improved piezoelectric performance, along with stable electrical responses under ultrasound stimulation. These combined characteristics provide a robust foundation for its application in promoting brain tissue repair and functional reconstruction.

The cytotoxicity of piezoelectric fibrous membranes was evaluated using a CCK‐8 (MCE, USA) assay. Sterilized materials (PLLA, CPLLA, and FF‐CPLLA) were co‐cultured with rat microglia for 24, 48, and 72 h. The results indicated minimal cytotoxicity of all materials toward microglia across all time points (Figure S17). After 48 h of co‐culture, live/dead staining was performed using calcein‐AM (green, live cells) and propidium iodide (red, dead cells). The FF‐CPLLA group showed no significant cell death and a relative increase in cell number (Figure S18A,B), demonstrating excellent biocompatibility of the piezoelectric fibrous membranes. Furthermore, to determine the optimal ultrasound stimulation intensity and exclude potential effects of ultrasound on cell viability, different ultrasound intensities were applied (0, 0.1, 0.3, 0.5, 0.7, 1.0, and 1.5 W/cm^2^; fixed parameters: 1 MHz, 50% duty cycle, 2 min) using an ultrasound coupling gel (Tianjin Jinya Technology Development Co., Ltd, China) applied to the outer bottom surface of the cell culture plate. It was observed that ultrasound intensities between 0.1 and 0.3 W/cm^2^ did not significantly affect cell viability, while intensities from 0.5 to 1.5 W/cm^2^ resulted in varying degrees of cell death (Figures S19 and S20). Based on these findings, the non‐cytotoxic FF‐CPLLA fibrous membranes and an optimized ultrasound parameter set (0.3 W/cm^2^, 1 MHz, 50% duty cycle, 2 min) were selected for subsequent experiments.

To further evaluate the effects of the material itself, ultrasound‐induced mechanical stimulation alone, and the piezoelectric effect of the ultrasound‐responsive fibrous membrane on cell viability, we conducted experiments based on preliminary safety profiles of the materials and previously optimized ultrasound parameters. Sterile FF‐CPLLA (strong piezoelectricity) and PLLA (weak piezoelectricity) fibrous membranes were cut into discs approximately 8 mm in diameter, placed in 48well plates, and cocultured with rat microglial cells. After cell attachment, the following groups were treated: I) Control, II) US, III) FF‐CPLLA, IV) US + PLLA, V) US + FF‐CPLLA. Ultrasound was applied at an intensity of 0.3 W/cm^2^ (1 MHz, 50% duty cycle, 2 min). Cell viability was assessed 12 h post‐stimulation using the CCK8 assay. Results showed that cell viability in the PLLA + US group (90.64% ± 4.557%) showed no significant difference compared with the Control, US, or FF‐CPLLA groups, but was significantly lower than that in the FF‐CPLLA + US group (110.3% ± 5.15%) (Figure 4A). This finding indicates that the enhanced cell viability observed in the FF‐CPLLA+US group is likely attributable to ultrasound‐activated piezoelectric stimulation, rather than to ultrasound exposure alone or purely mechanical vibration; nevertheless, a contribution from local acoustic–mechanical coupling effects cannot be entirely excluded.

**FIGURE 4:**
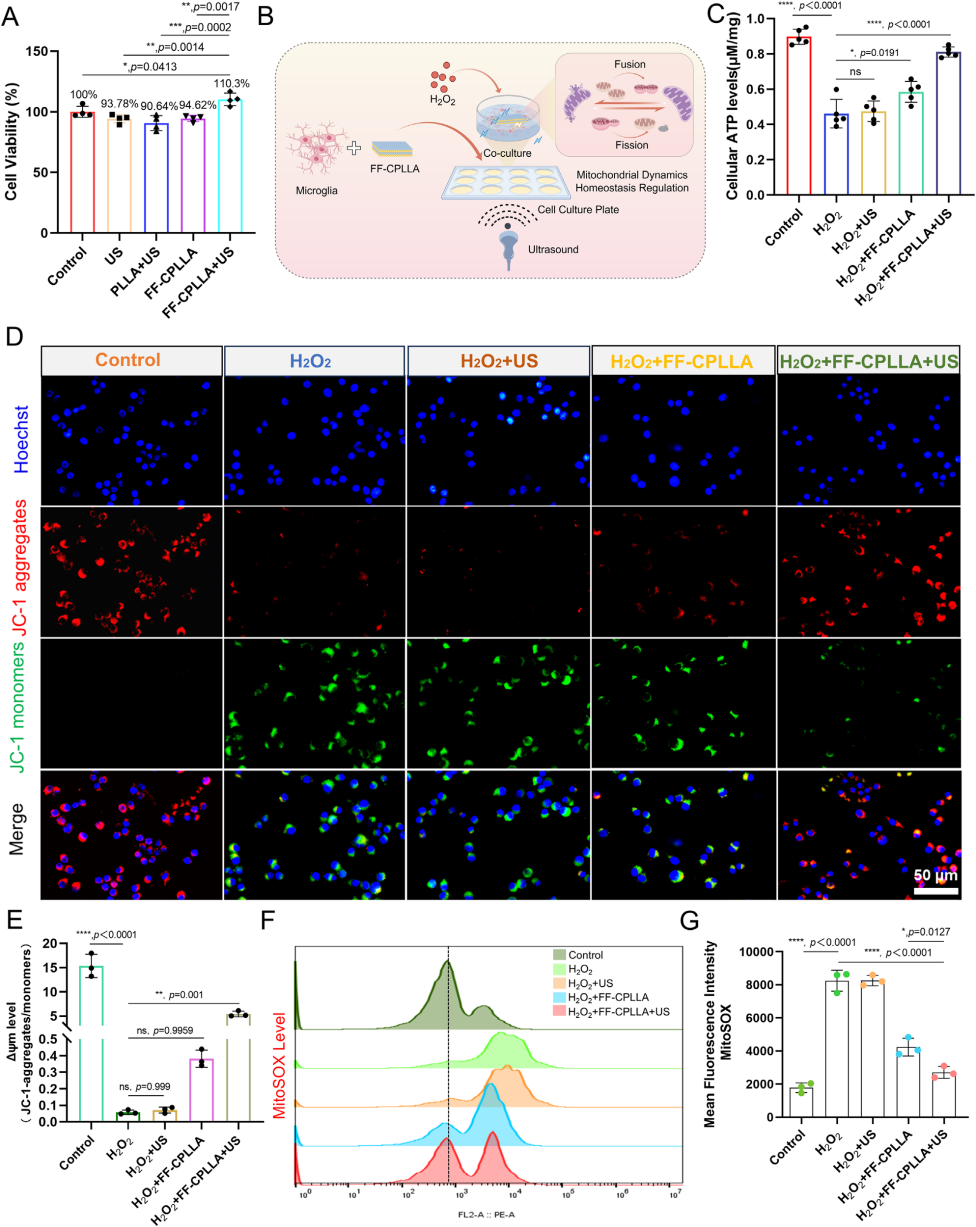
The ultrasound‐responsive FF‐CPLLA piezoelectric fibrous membrane ameliorates mitochondrial dysfunction and reduces mitochondrial superoxide production. (A) Effects of different interventions on the viability of rat microglial cells. Data are presented as mean ± SD; *n* = 4. (B) Schematic diagram illustrating the co‐culture of FF‐CPLLA with rat microglia and the experimental intervention protocol. (C) Intracellular ATP levels measured by an ATP assay kit across different treatment groups. Data are presented as mean ± SD; *n* = 5. (D) Representative fluorescent images of JC‐1 staining in different groups. JC‐1 aggregates and monomers were visualized in the red and green channels, respectively. Scale bar, 50 µm. (E) Quantitative analysis of the average fluorescence intensity of JC‐1 staining, reflecting the mitochondrial membrane potential. Data are presented as mean ± SD; *n* = 3. One‐way ANOVA was used for statistical analysis. (F) Measurement of mitochondrial superoxide levels in cells by flow cytometry. (G) Quantitative analysis of the mean fluorescence intensity from flow cytometry. Data are presented as mean ± SD; *n* = 3. One‐way ANOVA was used for statistical analysis.

### 2.2 Establishment of an in vitro Model of Mitochondrial Oxidative Stress Injury

We established an in vitro model of mitochondrial oxidative stress injury by stimulating rat microglia with different concentrations of H_2_O_2_. The optimal concentration and duration of H_2_O_2_ treatment were determined based on CCK‐8 assay and JC‐1 (MCE, USA) staining. Ultimately, treatment with 400 µM H_2_O_2_ for 12 h was selected, as it maintained microglial viability above 80% (Figure S21). JC‐1 staining combined with flow cytometry analysis revealed a significant decrease in mitochondrial membrane potential, with the JC‐1 monomer proportion reaching 20.4% (Figure S22), confirming the successful establishment of the mitochondrial oxidative stress injury model.

### 2.3 Ultrasound‐Responsive Piezoelectric Fibrous Membranes Modulate Mitochondrial Dynamics Homeostasis to Ameliorate Mitochondrial Dysfunction

Mitochondrial dysfunction is increasingly implicated as a key pathophysiological mechanism underlying a spectrum of disorders, such as neurodegenerative diseases, metabolic syndromes, and age‐related pathologies [26, 27, 28]. It drives disease progression primarily through energy depletion, exacerbated oxidative stress, and activation of cell death signaling pathways. Developing novel intervention strategies capable of effectively restoring mitochondrial function is of significant clinical importance. To evaluate the effect of piezoelectric stimulation on mitochondrial dysfunction in rat microglial cells, an oxidative stress injury model was established using H_2_O_2_ treatment (Figure 4B). The cells were divided into five experimental groups:I) Control, II) H_2_O_2_, III) H_2_O_2_ + US, IV) H_2_O_2_ + FF‑CPLLA, and V) H_2_O_2_ + FF‑CPLLA + US. Ultrasound stimulation was applied at an intensity of 0.3 W/cm^2^ (1 MHz, 50% duty cycle) for 2 min. The results showed that ATP synthesis decreased in H_2_O_2_‐stimulated microglia, indicating impaired mitochondrial oxidative phosphorylation. However, after treatment with FF‐CPLLA + US, intracellular ATP levels were restored, and the recovery effect was significantly better than that in the group with non‐ultrasound‐activated fibrous membranes (FF‐CPLLA) (Figure 4C). This phenomenon confirms that ultrasound‐responsive piezoelectric fibrous membranes can enhance mitochondrial energy metabolism through the piezoelectric effect, serving as a positive indicator of restored mitochondrial function [29]. Additionally, mitochondrial dysfunction is often accompanied by collapse of ΔΨm and excessive production of mitochondrial superoxide [30]. Using JC‐1 fluorescence staining and MitoSOX Red flow cytometry analysis, we verified that H_2_O_2_ stimulation indeed aggravated the loss of mitochondrial membrane potential and significantly increased mitochondrial superoxide levels. Importantly, after treatment with FF‐CPLLA + US, mitochondrial membrane potential was markedly restored, and mitochondrial superoxide expression was significantly reduced (Figure 4D–G). Together, these results demonstrate that ultrasound‐responsive piezoelectric fibrous membrane not only restores mitochondrial energy metabolism but also effectively alleviates mitochondrial oxidative stress and maintains mitochondrial membrane potential stability, thereby repairing mitochondrial dysfunction.

Mitochondrial functional recovery is closely associated with the dynamic balance of its morphological network [31]. Previous studies have indicated that when mitochondria are damaged or dysfunctional, their normal morphology and function are severely compromised, potentially leading to a range of diseases [32]. In this study, mitochondria in different groups were labeled with a mitochondrial red fluorescent probe, imaged using confocal microscopy, and subsequently processed and skeletonized with ImageJ software. The results indicated that H_2_O_2_ stimulation led to shortened mitochondrial branches and fragmented network changes, while FF‐CPLLA+US intervention significantly reversed these pathological alterations, significantly increasing both the mean mitochondrial branch length and the mitochondrial area. (Figure 5A–C). Given that mitochondrial dynamics (fission/fusion) directly regulate their morphology and function [33], and that imbalance in dynamics can exacerbate mitochondrial dysfunction [34], we further examined the expression of key regulatory proteins involved in fission and fusion. Western blot analysis revealed that FF‐CPLLA+US treatment significantly suppressed the expression of phosphorylated Drp1 (p‐Drp1^Ser616^) induced by H_2_O_2_, while upregulating the expression of the fusion protein MFN1 (Figure 5D–G). Immunofluorescence staining was further performed to examine the expression and distribution of p‐Drp1^(Ser616)^. The results revealed that H_2_O_2_ stimulation markedly enhanced the fluorescence signal of p‐Drp1^(Ser616)^ in cells, which showed evident colocalization with mitochondrial markers. In contrast, after FF‐CPLLA + US treatment, the overall fluorescence intensity of p‐Drp1^(Ser616)^ was significantly reduced, and its colocalization with mitochondria was also markedly diminished (Figure 5H,I). These findings suggest that the ultrasound‐responsive piezoelectric fibrous membrane likely suppresses excessive mitochondrial fission by upregulating the expression of the fusion protein MFN1, reducing the phosphorylation level of Drp1, and decreasing its recruitment to mitochondria.

**FIGURE 5:**
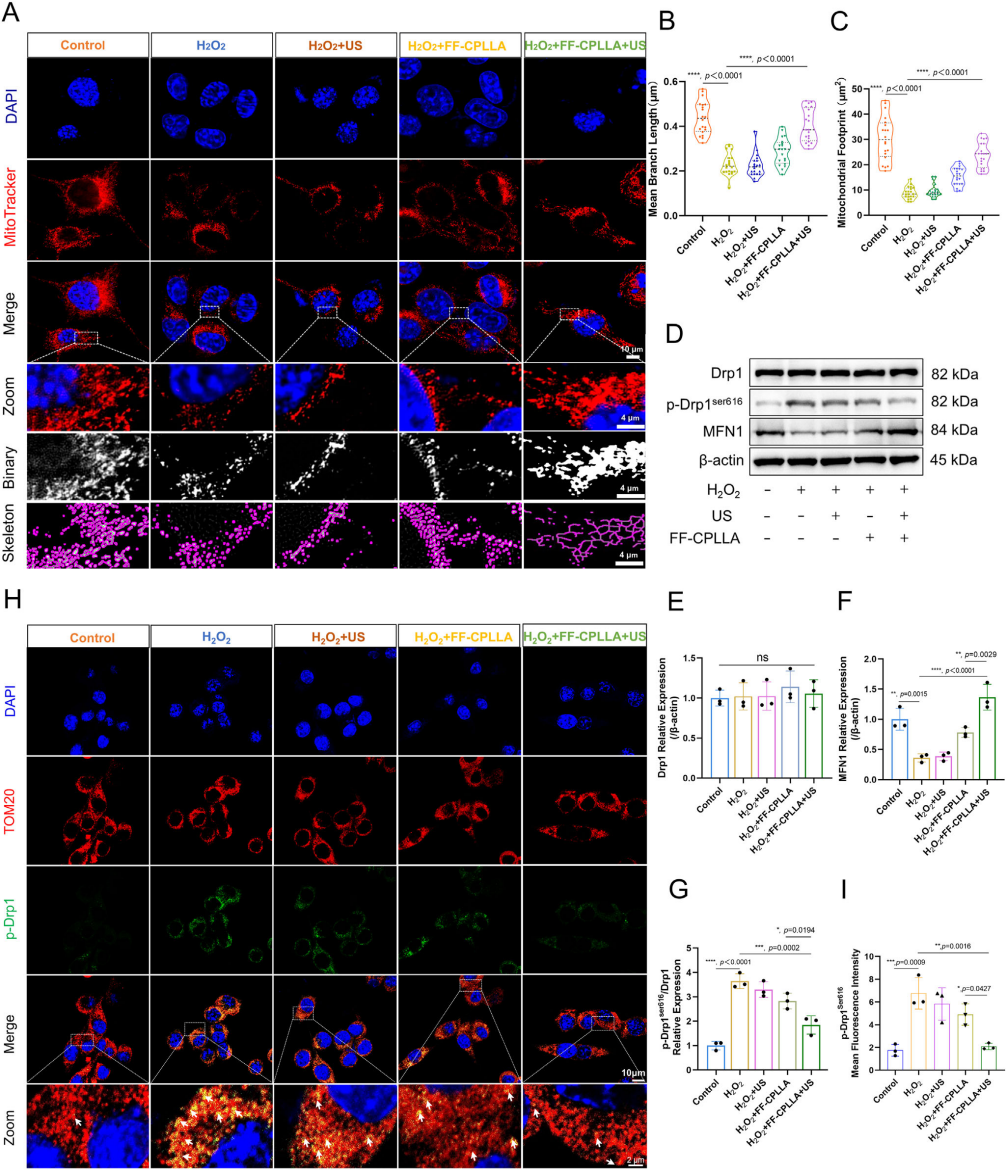
The ultrasound‐responsive FF‐CPLLA piezoelectric fibrous membrane regulates mitochondrial dynamics and improves mitochondrial network morphology. (A) Representative images of mitochondrial morphology in H_2_O_2_‐treated rat microglia, stained with MitoTracker Red and analyzed using the MiNA plugin in ImageJ. Scale bars: 10 µm (overview), 4 µm (enlarged views). (B,C) Statistical analysis of mitochondrial mean branch length and mitochondrial footprint data was performed. Data are presented as mean ± SD; *n* = 20. (D–G) Western blot analysis and corresponding quantification of Drp1, phospho‐Drp1 (p‐Drp1), and MFN1 protein expression levels across different groups. Data are presented as mean ± SD; *n* = 3. One‐way ANOVA was used for statistical analysis. (H) Immunofluorescence staining showing the co‐localization of TOM20 (red, mitochondrial outer membrane marker) and p‐Drp1 (green) in cells. Scale bars: 10 µm (overview), 2 µm (enlarged view). (I) Quantitative analysis of the mean fluorescence intensity p‐DRP1. Data are presented as mean ± SD; *n* = 3. One‐way ANOVA was used for statistical analysis.

Furthermore, to clarify the effects of the material itself and its combined action with ultrasound on mitochondrial dynamics, and to investigate whether the underlying mechanism originates from the physical properties of the material, the mechanical stimulation of ultrasound alone, or the synergistic piezoelectric effect elicited by their combination, rat microglial cells were divided into four groups under exogenous oxidative stress‐free conditions: I) Control, II) US, III) FF‐CPLLA, and IV) FF‐CPLLA+US. The ultrasound parameters were set to the previously determined safe conditions (0.3 W/cm^2^, 1 MHz, 50% duty cycle, duration 2 min). Analysis of mitochondrial dynamics‐related protein expression by Western blotting showed (Figure 6A–D), Compared to the Control group, ultrasound stimulation alone showed a trend of increasing the expression of the mitochondrial fission protein p‐Drp1^(Ser616)^, but the difference was not statistically significant. Moreover, this condition did not significantly alter the expression levels of the fusion proteins MFN1 and MFN2, suggesting that ultrasound at this intensity by itself does not disrupt the molecular balance of mitochondrial fission and fusion under physiological conditions. In cells cultured solely on the FF‐CPLLA fibrous membrane, the expression of both MFN1 and MFN2 showed a slight upregulation, indicating that this material, as a physical microenvironmental cue, can exert a regulatory influence on the mitochondrial network under cellular homeostasis, favoring a shift toward fusion. Notably, the combined FF‐CPLLA+US treatment demonstrated a clear synergistic regulatory effect. Compared to the material‐alone group, the combined treatment further significantly upregulated the protein expression levels of both MFN1 and MFN2. This suggests that its regulatory effect is likely not a simple additive result of the material and ultrasound actions, but may stem from a unique biological response triggered by their synergy.

**FIGURE 6:**
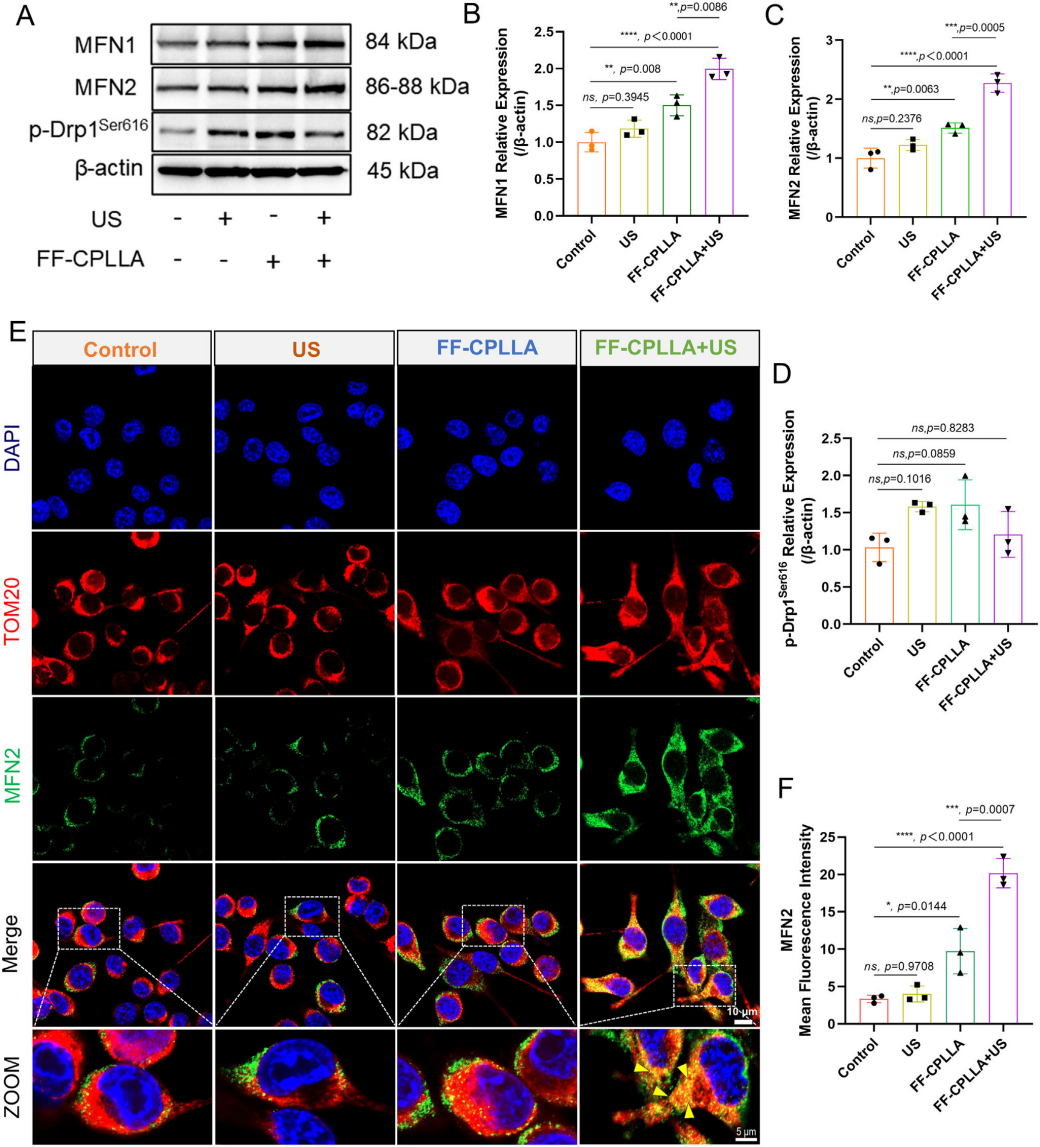
Effects of interventions on mitochondrial dynamics in rat microglial cells. (A–D) Western blot analysis and quantification of p‐Drp1, MFN1, and MFN2 protein expression levels in different groups. Data are presented as mean ± SD (*n* = 3). (E) Immunofluorescence staining showing the co‐localization of TOM20 (red, mitochondrial outer membrane marker) and MFN2 (green) in cells. Scale bars: 10 µm (overview), 5 µm (enlarged view). (F) Quantitative analysis of the mean fluorescence intensity MFN2. Data are presented as mean ± SD; *n* = 3. One‐way ANOVA was used for statistical analysis.

We further examined the expression and distribution of MFN2 within mitochondria via immunofluorescence staining. The results showed that in the FF‐CPLLA+US group, MFN2 fluorescence intensity was markedly enhanced and exhibited significantly stronger colocalization with mitochondrial markers (Figure 6E,F). As a key executor of mitochondrial outer membrane fusion, the increased mitochondrial localization of MFN2 indicates effective activation of the fusion machinery. These findings further demonstrate that ultrasound‐responsive piezoelectric stimulation not only upregulates MFN2 protein expression but, more importantly, promotes the enrichment of MFN2 to the mitochondrial outer membrane, thereby establishing a functional basis for the membrane fusion process it mediates.

### 2.4 Ultrasound‐Responsive Piezoelectric Fibrous Membranes Ameliorate Abnormal Opening of the Mitochondrial Permeability Transition Pore (MPTP)

The mitochondrial permeability transition pore (MPTP) is defined as a non‐specific channel spanning the mitochondrial membranes. Its pathological, sustained opening acts as a key mediator of dysfunction, resulting in mitochondrial swelling and loss of membrane potential [35]. Using the Calcein‐AM/CoCl_2_ quenching assay, we evaluated the effect of piezoelectric stimulation on mitochondrial MPTP opening in cells. We found that ultrasound‐responsive piezoelectric fibrous membrane effectively ameliorated the abnormally sustained activation of MPTP induced by H_2_O_2_ (Figure 7A,B). These results confirm that ultrasound‐responsive piezoelectric fibrous membranes not only restore mitochondrial energy metabolism and redox balance but also directly preserve mitochondrial membrane integrity and inhibit the pathological response of MPTP. The opening of MPTP is regulated by multiple factors, including mitochondrial calcium overload, oxidative stress, and changes in mitochondrial membrane potential [36]. Notably, this study found that ultrasound‐responsive piezoelectric fibrous membrane improved mitochondrial membrane potential and reduced mitochondrial superoxide accumulation. We speculate that the locally generated micro‐electric field from piezoelectric stimulation may directly influence mitochondrial membrane potential or coordinate with ion channels to mitigate oxidative stress, thereby indirectly suppressing MPTP opening. However, this mechanism requires further investigation for validation.

**FIGURE 7:**
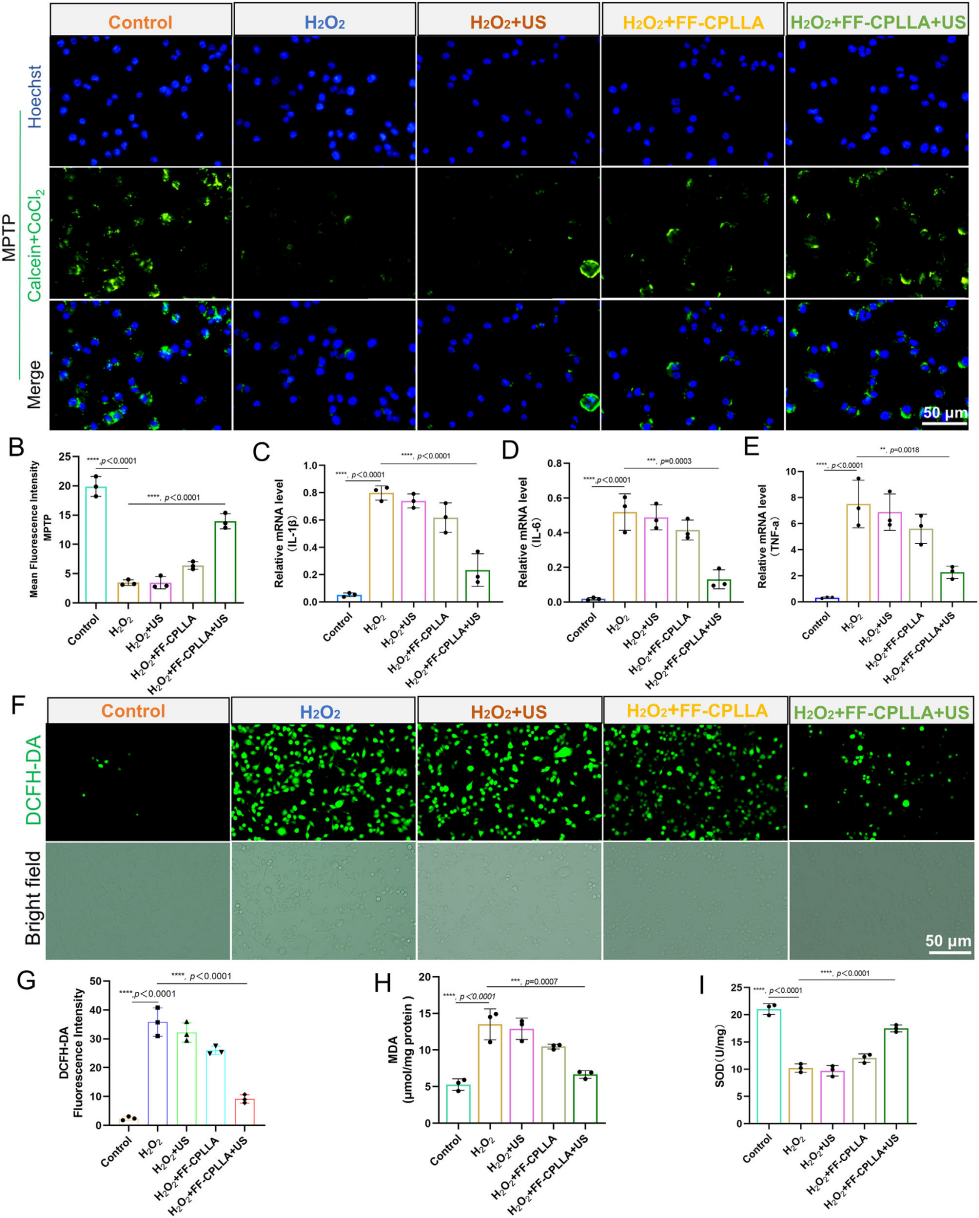
The ultrasound‐responsive FF‐CPLLA piezoelectric fibrous membrane ameliorates MPTP opening, neuroinflammation, and oxidative stress. (A) Representative fluorescence images showing the degree of MPTP opening in different groups, detected by an MPTP assay kit. Scale bar, 50 µm. (B) Quantitative analysis of the mean fluorescence intensity reflecting MPTP opening. Data are presented as mean ± SD; *n* = 3. One‐way ANOVA was used for statistical analysis. (C–E) mRNA expression levels of pro‐inflammatory cytokines (IL‐1β, IL‐6, TNF‐α) measured by RT‐qPCR in different groups. Data are presented as mean ± SD; *n* = 3. One‐way ANOVA was used for statistical analysis. (F) Representative fluorescence images of intracellular ROS detected by the DCFH‐DA probe. Scale bar, 50 µm. (G) Quantitative analysis of the mean fluorescence intensity of DCFH‐DA. Data are presented as mean ± SD; *n* = 3. One‐way ANOVA was used for statistical analysis. (H, I) Levels of MDA and SOD activity measured by commercial assay kits in different groups. Data are presented as mean ± SD; *n* = 3. One‐way ANOVA was used for statistical analysis.

### 2.5 Simultaneous Reversal of the Oxidative Stress‐Inflammation Cascade

Mitochondrial dysfunction is not only the root cause of disrupted energy metabolism but also a central hub driving the vicious cycle of oxidative stress and inflammation [11]. Previous studies have shown that gold nanoparticle‐modified piezoelectric nanomaterials (Au@BT) exhibit significant synergistic anti‐inflammatory effects and promote the recovery of motor function in rats with spinal cord injury [37]. This study confirmed that H_2_O_2_ stimulation triggered a significant increase in the gene expression of inflammatory factors (IL‐1β, IL‐6, TNF‐α) in rat microglial cells, accompanied by the accumulation of ROS and MDA, as well as a decrease in SOD activity. However, treatment with FF‐CPLLA+US not only suppressed the expression of inflammatory genes in rat microglial cells but also significantly reduced ROS and MDA levels while enhancing SOD activity (Figures 7C,I). These findings suggest that ultrasound‐responsive piezoelectric fibrous membrane may disrupt the vicious cycle of oxidative stress and inflammation by restoring mitochondrial function. Previous research has indicated that mitochondrial dysfunction can activate the NLRP3 inflammasome, leading to the cleavage of substrates such as Pro‐IL‐1β, promoting their maturation and extracellular release, thereby exacerbating inflammatory responses [38]. Further mechanistic investigation revealed that ultrasound‐responsive piezoelectric fibrous membrane significantly suppressed the expression of NLRP3 inflammasome proteins (Figure 8A,B). Based on previous studies [39, 40], we hypothesize that ultrasound‐responsive piezoelectric fibrous membrane helps restore mitochondrial network homeostasis and stabilizes the MPTP, thereby reducing excessive ROS production from damaged mitochondria and preventing the leakage of mitochondrial damage‐associated molecular patterns (such as mtDNA and ROS). This process may indirectly attenuate inflammasome assembly signaling and contribute to synergistic anti‐inflammatory effects.

**FIGURE 8:**
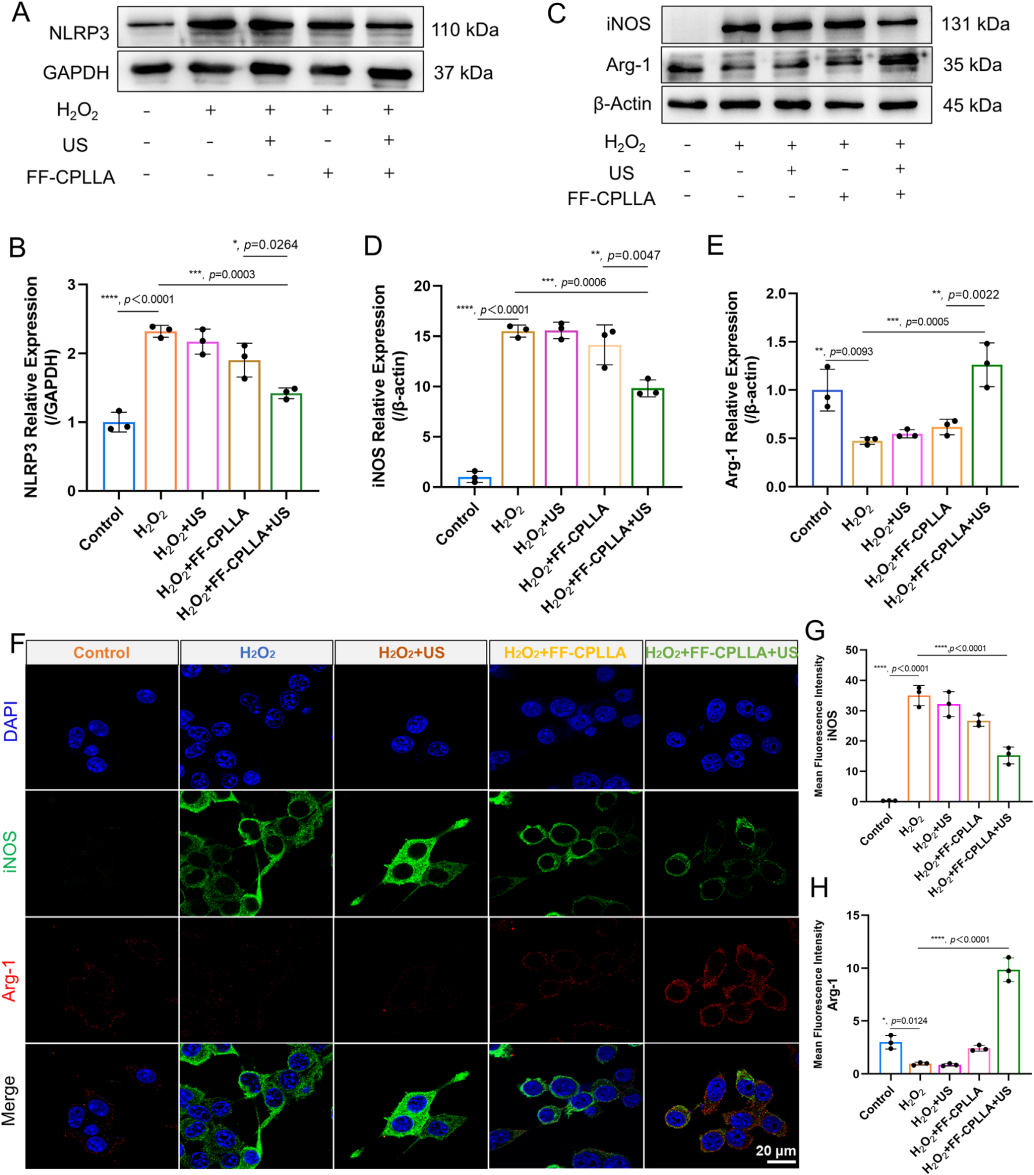
The ultrasound‐responsive FF‐CPLLA piezoelectric fibrous membrane reduces NLRP3 inflammasome protein expression and promotes microglial polarization towards the M2 phenotype. (A, B) Western blot analysis and corresponding quantification of NLRP3 protein expression levels across different groups. Data are presented as mean ± SD; *n* = 3. One‐way ANOVA was used for statistical analysis. (C–E) Western blot analysis and quantification of iNOS and Arg‐1 protein expression levels in different groups. Data are presented as mean ± SD; *n* = 3. One‐way ANOVA was used for statistical analysis. (F) Immunofluorescence staining showing the expression and co‐localization of iNOS (green) and Arg‐1 (red) in cells. Scale bar, 20 µm. (G, H) Quantitative analysis of the mean fluorescence intensity for iNOS and Arg‐1. Data are presented as mean ± SD; *n* = 3. One‐way ANOVA was used for statistical analysis.

### 2.6 Ultrasound‐Responsive Piezoelectric Fibrous Membranes Promote Polarization of Rat Microglial Cells Toward the M2 Phenotype

In recent years, immune cell‐based engineering strategies have emerged as a pivotal direction in immunotherapy. These approaches utilize nano‐ and genetic engineering techniques to harness the inherent properties of cells for modulating the local immune microenvironment [41]. Microglia, as central immune regulators in the central nervous system, directly influence the processes of neural injury and repair through their phenotypic polarization states (M1 and M2 types) [42]. Overactivation of M1‐type microglia can lead to neuronal dysfunction, injury, and degeneration, whereas M2‐type microglia exert neuroprotective effects by promoting tissue repair and regeneration. Previous studies have shown that piezoelectric stimulation can induce M2 polarization in macrophages, reconstruct a regenerative immune microenvironment, and facilitate periodontal stem cell regeneration [43]. Chen et al. [44] systematically elaborated on how nanomaterials enable the noninvasive labeling, in situ programming, and long‐term functional persistence of immune cells in vivo, showcasing the immense potential of materials engineering in precision immunomodulation. We applied piezoelectric stimulation to H_2_O_2_‐induced rat microglial cells to investigate its effect on their phenotypic polarization. Protein expression of polarization markers was assessed using Western blot. After H_2_O_2_ induction, the protein level of the M1 marker iNOS was significantly increased. Interestingly, after FF‐CPLLA+US treatment, the expression of iNOS was downregulated, while the expression of the M2 marker Arg‐1 was increased. However, no significant differences were observed between the H_2_O_2_+US, H_2_O_2_+FF‐CPLLA groups and the H_2_O_2_ alone group (Figure 8C,E). These findings were further confirmed by immunofluorescence staining, which yielded consistent results (Figure 8F–H). Together, these data demonstrate that ultrasound‐responsive piezoelectric fibrous membrane promotes the polarization of microglia toward the M2 phenotype. M2 microglia have been shown to secrete anti‐inflammatory factors and neurotrophic factors [45, 46]. This effect, combined with the reduction of oxidative damage induced by piezoelectric stimulation, may provide dual protective mechanisms for neurons.

### 2.7 Study on the Promotion of Brain Tissue Repair in Rats Following TBI by Piezoelectric Fibrous Membranes

To systematically evaluate the neurorestorative effects of the ultrasound‐responsive piezoelectric fibrous membrane on traumatic brain injury (TBI) at the in vivo level, a rat controlled cortical impact (CCI) model was established. Post‐surgery, animals in the treatment groups were implanted with an FF‑CPLLA fibrous membrane (approximately 0.8 × 0.8 cm) according to the experimental design. Owing to its excellent flexibility and tissue conformability, similar to the structural advantages demonstrated by flexible electronic materials for neural interfaces as reported by Hou et al. [47], the membrane closely adheres to the brain surface and fully covers the cranial window (approximately 0.5 × 0.5 cm) and its surrounding area, thereby forming a stable and seamless physical interface (Figure 9A,B). Furthermore, during the acute phase following TBI, to accommodate brain tissue swelling caused by elevated intracranial pressure, the dura was not sutured after membrane placement. This procedure allowed for compensatory expansion of the injured brain tissue, which helped mitigate secondary cerebral edema, prevent brain herniation, and simultaneously enabled the material to function as a piezoelectric stimulation interface while adapting to and buffering acute pathophysiological changes. Rats were randomly assigned to five groups: Sham, TBI, TBI + US, TBI + FF‑CPLLA, and TBI + FF‑CPLLA + US. Ultrasound stimulation was administered once daily (at 9:00 a.m.) using the following parameters: 0.3 W/cm^2^, 1 MHz, 50% duty cycle, 2 min per session.

**FIGURE 9:**
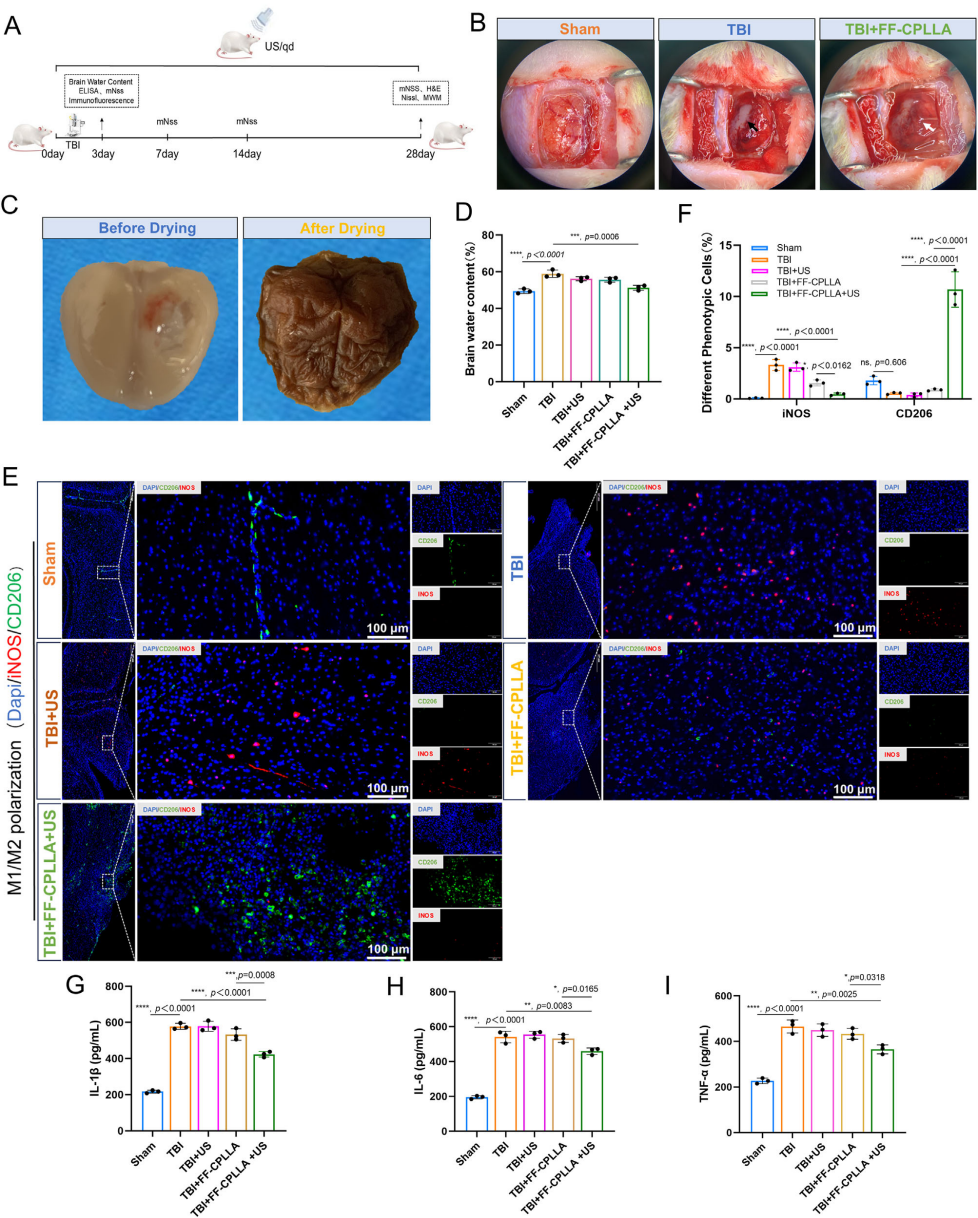
Ultrasound‐responsive FF‐CPLLA piezoelectric fibrous membrane alleviates cerebral edema and neuroinflammation, and promotes the expression of the M2 phenotype marker CD206 in brain tissue. (A) Animal model and intervention timeline. (B) Large craniectomy for rat TBI model and in situ application of piezoelectric material. Black arrow: core injury area; white arrow: in situ application of FF‐CPLLA. (C) Representative images showing rat brain tissue before and after drying at 110°C. (D) Detection and quantification of brain water content in rats. Data are presented as mean ± SD; *n* = 3. One‐way ANOVA was used. (E,F) Fluorescence co‐staining images and quantification of iNOS and CD206 in rat brain tissue sections. Scale bar: 100 µm. Data are presented as mean ± SD; *n* = 3. One‐way ANOVA was used. (G–I) Expression of inflammatory cytokines IL‐1β, IL‐6, and TNF‐α in brain tissue homogenate supernatant detected by ELISA kits. Data are presented as mean ± SD; *n* = 3. One‐way ANOVA was used.

Acute cerebral edema is closely associated with poor functional outcomes [48]. Previous studies have indicated that targeted treatment addressing the underlying causes of post‐TBI cerebral edema can significantly improve functional recovery in mice after TBI [49]. We measured changes in brain water content in rats 3 days after TBI and found that compared to the TBI‐only group, the TBI + FF‐CPLLA + US treatment group exhibited a significant reduction in cerebral edema (Figure 9C,D). One of the primary contributors to post‐traumatic cerebral edema is cytotoxic damage resulting from alterations in the immune microenvironment [50]. To further validate whether piezoelectric stimulation can rebalance microglial phenotypes and restore the local immune microenvironment to promote neural repair, brain tissues were collected and fixed 3 days after TBI for immunofluorescence analysis. The expression of the M1 phenotype marker iNOS was significantly increased in the TBI‐injured area. After piezoelectric stimulation treatment, iNOS expression decreased, while the expression of the M2 phenotype marker CD206 was markedly elevated (Figure 9E,F). Additionally, we used ELISA kits to measure the expression of inflammatory factors in the brain tissue. The results showed that the local immune microenvironment in the TBI‐injured area was disrupted, with elevated levels of inflammatory factors. ultrasound‐responsive piezoelectric fibrous membrane treatment effectively reduced the expression levels of IL‐1β, IL‐6, and TNF‐α in the acute phase post‐TBI (Figure 9G–I).

Overactivated microglia serve as a central driver of neuroinflammation in the central nervous system (CNS). Their dynamic interplay with changes in the immune microenvironment collectively underlies the key pathological mechanisms of various neurological disorders [51]. These hyperactivated microglia release large amounts of pro‐inflammatory cytokines, which can directly damage neurons and oligodendrocytes, or further activate other CNS cells to produce additional inflammatory factors, thereby initiating an inflammatory cascade. We used IBA‐1 to label activated microglia in brain tissue and found a significant increase in activated microglia in the injured area following TBI. However, after treatment with ultrasound piezoelectric stimulation, IBA‐1 expression was markedly reduced (Figure 10A,B). Consistent with the findings of Wang et al. [52], this result fully demonstrates that ultrasound‐responsive piezoelectric fibrous membrane alleviates the overactivation of microglia. We used transmission electron microscopy to observe morphological changes in mitochondria in brain tissue sections. In the sham‐operated group, mitochondria exhibited normal size and intact morphology with well‐organized structures. In the TBI group, however, mitochondria showed extensive swelling and rounding, disrupted and fragmented cristae, internal vacuolation, ruptured and discontinuous outer membranes leading to content leakage, and a significant increase in mitochondrial fission. Following ultrasound‐responsive piezoelectric fibrous membrane treatment, we observed that compared to the TBI group, mitochondrial swelling and rounding were significantly alleviated. Mitochondria exhibited a more elongated/rod‐like shape with relatively clear contours. The number and density of mitochondrial cristae showed notable recovery compared to the TBI group, with reduced cristae fragmentation, dissolution, and internal vacuolation. Partial mitochondrial fusion was also observed (Figure 10C). These results strongly indicate that ultrasound‐responsive piezoelectric stimulation contributes to mitochondrial repair.

**FIGURE 10:**
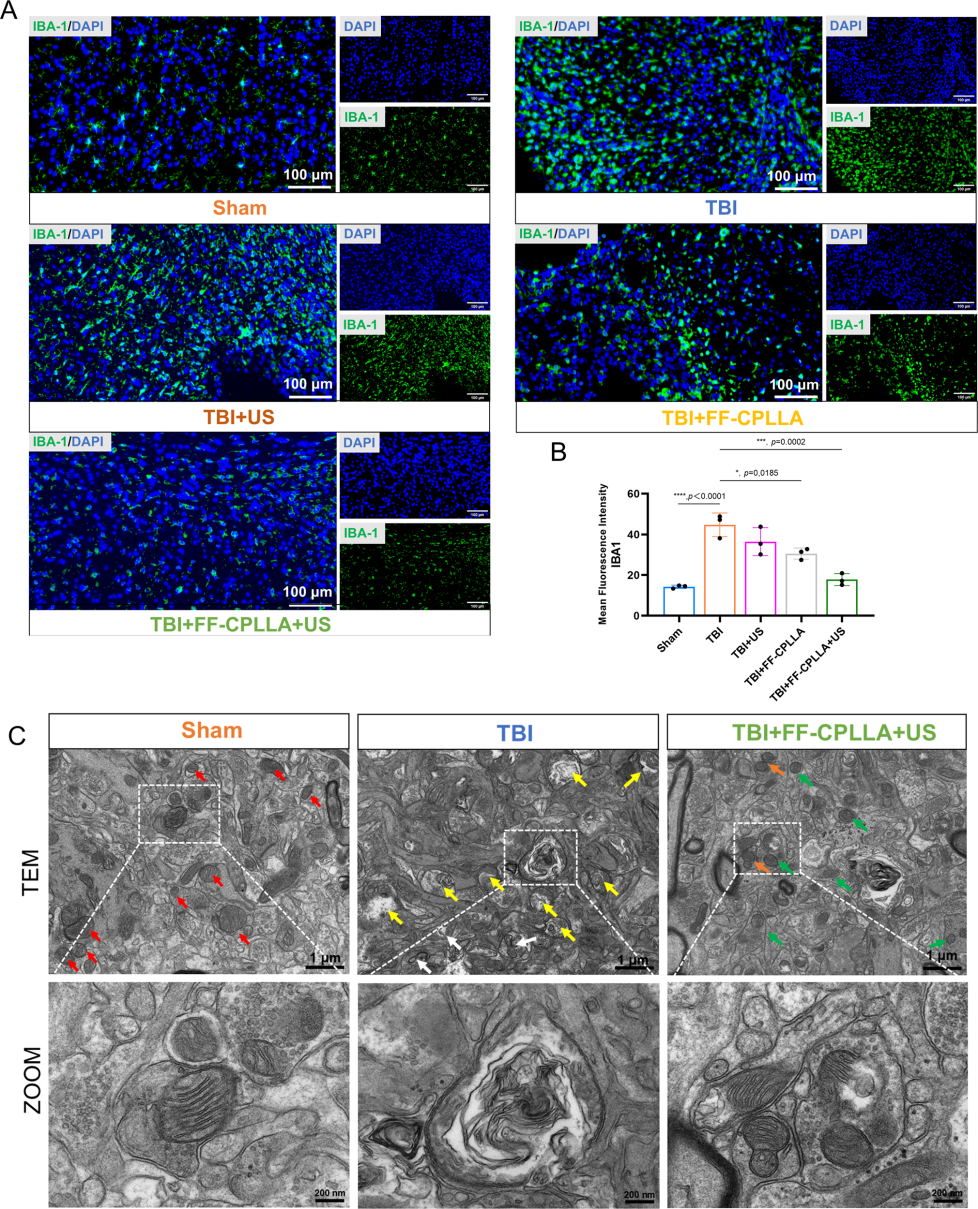
Ultrasound‐responsive FF‐CPLLA piezoelectric fibrous membrane reduces excessive activation of microglia and improves mitochondrial morphology and function after TBI. (A,B) Fluorescence staining images (A) and quantification (B) of IBA‐1 in rat brain tissue sections. Scale bar, 100 µm. Data are presented as mean ± SD; *n* = 3. One‐way ANOVA was used. (C) Morphological changes of mitochondria in brain tissue observed under transmission electron microscopy. Red arrows: normal mitochondria; Yellow arrows: swollen mitochondria with cristae fragmentation and vacuolation; White arrows: mitochondrial fission; Green arrows: mitochondria showing a trend towards normal morphology; Orange arrows: mitochondrial fusion. Scale bars,  1 µm and 200 nm.

The restoration of the immune microenvironment provides crucial support for neural repair following trauma [53]. We assessed neurological functional changes in rats at 3, 7, 14, and 28 days after TBI using the Modified Neurological Severity Score (mNSS). The TBI + FF‑CPLLA + US group showed significantly better functional recovery compared to the TBI group from day 3 onward (Figure 11A). At 28 days post‐TBI, the Morris water maze test was further employed to evaluate learning and memory abilities. During the 5‐day training period, the escape latency of the rats gradually shortened, which serves as a key indicator of learning capacity. Compared with the TBI group and the TBI + US group, the TBI + FF‐CPLLA + US group showed significantly shorter escape latency over the 5‐day training period (Figure 11B), indicating that ultrasound‐responsive piezoelectric fibrous membrane enhanced learning ability, which may be associated with the recovery of neurological function. On day 6, the platform was removed to assess memory retention. The movement trajectories of each group are shown in Figure 11C. Rats in the TBI + FF‐CPLLA + US group were better able to remember the location of the platform, whereas the TBI group exhibited more disorganized swimming paths. The percentage of time spent in the target quadrant (% Time in Target Quadrant) and the number of crossings over the original platform location further demonstrated that ultrasound‐responsive piezoelectric fibrous membrane promoted the recovery of memory function (Figure 11D,E). We also compared swimming speeds among the groups and found no significant differences, indicating that motor performance did not influence the behavioral outcomes (Figure 11F). These findings closely align with the central objective of current biomaterial scaffold research, which aims to promote neural circuit remodeling and functional recovery by providing a supportive microenvironment [54].

**FIGURE 11:**
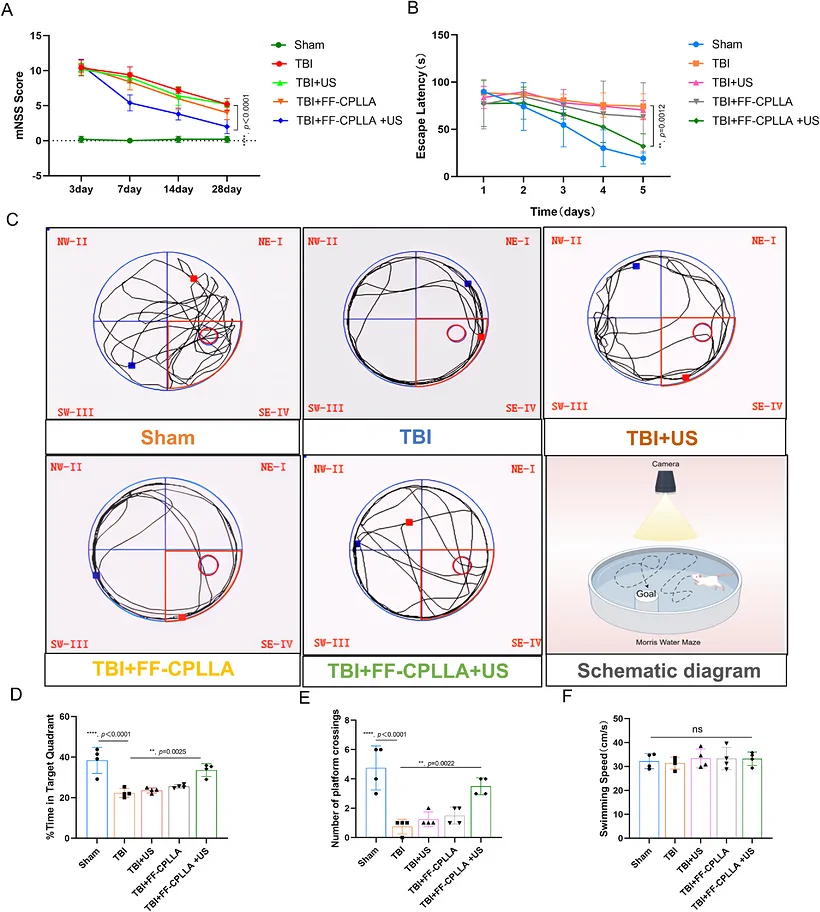
Ultrasound‐responsive FF‐CPLLA piezoelectric fibrous membrane promotes neurological functional recovery and improves learning and memory function in rats after TBI. (A) mNSS scores of TBI rats at different time points. Data are presented as mean ± SD; *n* = 5. One‐way ANOVA was used. (B) Escape latency to the platform for rats in each group. Data are presented as mean ± SD; *n* = 4. One‐way ANOVA was used. (C) Representative movement trajectories of rats in each group and Schematic diagram of the Morris water maze test. (D) Time spent in the target quadrant by rats in each group. Data are presented as mean ± SD; *n* = 4. One‐way ANOVA was used. (E) Number of platform crossings by rats in each group. Data are presented as mean ± SD; *n* = 4. One‐way ANOVA was used. (F) Average swimming speed of rats in each group. Data are presented as mean ± SD; *n* = 4. One‐way ANOVA was used.

Twenty‐eight days after TBI surgery, brain tissues were collected and subjected to H&E staining to observe cortical integrity and morphological changes in neural cells. As shown in Figure 12A,B, the cerebral cortex in the TBI and TBI + US groups exhibited significant disruption, with lesions extending into the hippocampus, while the TBI + FF‐CPLLA + US group showed markedly reduced areas of damage. In the Sham group, cortical neurons were arranged orderly with regular and dense morphology. In contrast, the TBI and TBI+US groups displayed disorganized cortical neuronal architecture accompanied by extensive necrosis. In the TBI + FF‐CPLLA + US group, neuronal necrosis was alleviated, and the arrangement and morphology of neural cells in the injured area tended to normalize. These findings further indicate that ultrasound‐responsive piezoelectric fibrous membrane promotes neural repair and reduces neuronal necrosis. We further applied Nissl staining to assess neuronal damage. After neuronal injury, the number of Nissl bodies decreases or even disappears. As shown in Figure 12C, the TBI group exhibited disorganized Nissl body structure with extensive loss, replaced by proliferating fibrous tissue. In contrast, the TBI + FF‐CPLLA + US group showed a relative increase in Nissl bodies with more regular arrangement, further supporting that ultrasound‐responsive piezoelectric fibrous membrane mitigates neuronal necrosis and promotes neural recovery after TBI.

**FIGURE 12:**
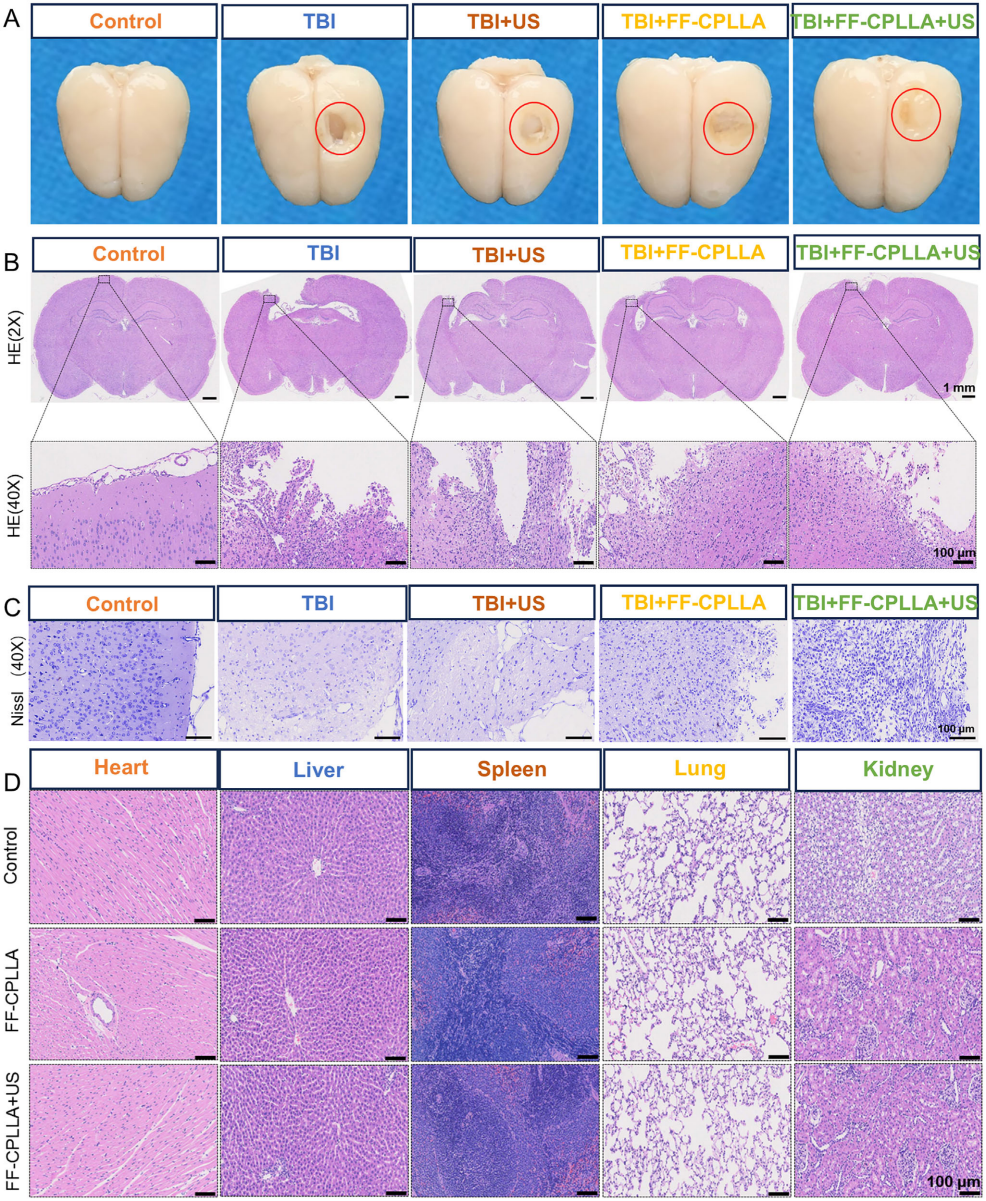
The ultrasound‐responsive FF‐CPLLA piezoelectric fibrous membrane alleviates brain tissue necrosis, promotes neural repair, and exhibits favorable biocompatibility. (A) Recovery of brain tissue injury in rats from different groups at 28 days post‐TBI. (B) H&E staining of brain tissue sections. Scale bars, 1 mm and 100 µm. (C) Nissl staining of brain tissue sections. Scale bar, 100 µm. (D) H&E staining of heart, liver, spleen, lung, and kidney tissues. Scale bar, 100 µm.

To further elucidate the therapeutic mechanism of the piezoelectric fibrous membrane at the whole‐transcriptome level, we performed RNA sequencing of the peri‐injury cortical tissue collected at postoperative day 3 from the Sham group, TBI model group (TB), and TBI + FF‐CPLLA + US treatment group (Tre) (*n* = 5). Principal component analysis (PCA) revealed a clear separation between the TB and Sham groups in transcriptional space, whereas the Tre group shifted markedly toward the Sham group (Figure S23A). Consistently, hierarchical clustering of differentially expressed genes (DEGs) demonstrated that the expression profile of the Tre group clustered together with that of the Sham group and was distinctly separated from that of the TB group (Figure S24B). Compared with the Sham group, a total of 3712 DEGs were identified in the TB group (2784 up‐regulated and 928 down‐regulated; |log2(FoldChange)|≥1 & padj≤0.05), whereas the number of DEGs between the Tre and Sham groups dropped to 1365. Direct comparison between the TB and Tre groups yielded only 126 DEGs (99 up‐regulated and 27 down‐regulated) (Figure S23C–E). These results quantitatively demonstrate that piezoelectric stimulation markedly attenuated the global transcriptional dysregulation induced by TBI. Gene Ontology (GO) enrichment analysis of these 126 DEGs revealed that they were predominantly enriched in biological processes such as “mitochondrial fission, ” “mitochondrial fusion, ” “mitochondrial protein quality control, ” and “inflammatory response” (Figure S24). Kyoto Encyclopedia of Genes and Genomes (KEGG) pathway enrichment analysis further showed that these DEGs were significantly enriched in the NOD‐like receptor signaling pathway and TNF signaling pathway (Figure S25), suggesting that this pathway is activated after TBI and suppressed by piezoelectric treatment. These transcriptomic alterations are highly consistent with our observations at the tissue level, including the down‐regulation of NLRP3 inflammasome protein (Figure S26A,B), the reduction of pro‐inflammatory cytokines (IL‐1β, IL‐6, TNF‐α) (Figure 9G–I), and the ultrastructural restoration of mitochondria (Figure 10C). Collectively, the whole‐transcriptome evidence confirms at the molecular network level that piezoelectric stimulation can systematically disrupt the vicious cycle of oxidative stress and inflammation driven by mitochondrial dysfunction after TBI, thereby providing a critical molecular foundation for neural repair.

At the experimental endpoints (7, 14, and 28 days post‐operation), the physical state and tissue response of the implanted FF‐CPLLA fibrous membrane were directly examined. The results demonstrated that at all time points, the membrane completely covered the injury area, remained in its original position, and showed no displacement. No adhesive encapsulation or significant inflammatory pseudomembrane formation was observed between the material and the surrounding brain tissue (Figure S27). Notably, over time, the membrane gradually integrated tightly with the adjacent skull and tissues, forming a sealed, dura‐like covering structure over the lesion area. In a separate experiment, FF‐CPLLA membranes were implanted on the brain surface of healthy rats and subjected to 28 days of ultrasound stimulation. H&E staining analysis of the implantation site (Figure S28) revealed that, compared to the sham group, neither the FF‐CPLLA membrane alone nor the combination with ultrasound stimulation (FF‐CPLLA+US) induced significant abnormal inflammatory cell infiltration or structural damage to the brain tissue. These findings indicate that within the 28‐day observation window of this study, the FF‐CPLLA fibrous membrane can function as a stable, biocompatible physical interface and a potential source of sustained piezoelectric stimulation, providing continuous support for the repair of the injured region. Furthermore, we evaluated the biosafety of the piezoelectric thin film through blood biochemical analysis (Table S2) and H&E staining of major organs (Figure 12D). Compared with the sham group, no significant tissue damage or morphological changes were observed in the major organs of any experimental group, indicating that the piezoelectric fibrous membrane exhibits no apparent biotoxicity.

Regarding the in vivo degradation, the fibrous membrane remained macroscopically intact with no observable structural disintegration throughout the study period (up to day 28) (see Figure S26). However, we noted a dynamic change in the mechanical properties of the material over time. At 7 days post‐implantation, the membrane retained relatively good integrity and could be peeled from the brain surface with relative completeness. By 14 days, the membrane had tightly integrated with the surrounding tissue, and its toughness demonstrated a trend of reduction. At 28 days post‐implantation, due to the tight integration and growth of the membrane with the adjacent tissue, attempts at retrieval using instruments revealed a significant decrease in toughness, making the membrane prone to fragmentation rather than allowing for relatively intact peeling as in the initial implantation stage (Figure S29). This suggests that the material may have undergone early‐stage hydrolysis or structural weakening, a phenomenon consistent with the initial degradation process of PLLA.

The FF‐CPLLA piezoelectric fibrous membrane developed in this study demonstrated excellent biocompatibility, interfacial stability, and promising neuro‐reparative potential over the 28‐day observation period. From a translational perspective, the slow degradation of its PLLA matrix offers distinct advantages during the critical neural repair window spanning weeks to months, as it provides durable physical support and sustained piezoelectric stimulation. However, complete degradation of PLLA requires several months to years, and its prolonged intracranial retention may pose potential risks such as chronic inflammation or interference with neural remodeling processes. Although our 28‐day observations confirm initial safety, they are insufficient to evaluate the full degradation profile or long‐term biological effects. Consequently, future translational efforts must prioritize aligning material degradation kinetics with the neural repair timeline. This will require not only comprehensive, long‐term safety assessments covering the entire degradation cycle but also the application of material engineering strategies including controlled polymer blending (e.g., with PCL), surface modification, and crystallinity regulation to precisely program degradation rates, ultimately leading to the development of intelligent neural interface materials that integrate optimal electroactivity with absorbable properties.

With regard to the therapeutic mechanism, the multi‐group control strategy employed in this study provides strong evidence that piezoelectric output is a necessary contributor to the observed therapeutic effects; however, demonstrating its sufficiency in complete isolation remains technically challenging, given the inherent physical coupling between the ultrasound field and the piezoelectric material. Under ultrasound exposure, local mechanical forces, microscale acoustic streaming, and piezoelectric charge generation are fundamentally intertwined. Therefore, while the collective evidence including the weak‐piezoelectric PLLA control, temperature monitoring, and direct electrical‐output measurements strongly supports ultrasound‐activated piezoelectric stimulation as the primary mechanism, the possibility that these physically coupled effects act in concert to produce the full therapeutic outcome cannot be excluded. This limitation in mechanistic dissection represents a shared challenge in the field of ultrasound‐responsive biomaterials and should be addressed in future studies through complementary approaches such as cavitation‐inhibitor controls or multiphysics computational modeling. Collectively, this study establishes a significant proof‐of‐concept foundation for these future advances in both materials engineering and mechanistic exploration.

## 3 Conclusion

This study designed an ultrasound‐responsive piezoelectric fibrous membrane (FF‐CPLLA). Under low‐intensity pulsed ultrasound (LIPUS) excitation, the fibrous membrane generates controllable electrical signals directly at the traumatic brain injury (TBI) lesion site, thereby selectively repairing mitochondrial dysfunction in microglia. Specifically, this treatment effectively restores mitochondrial dynamic homeostasis, stabilizes the mitochondrial membrane potential (ΔΨm), reduces excessive mitochondrial superoxide accumulation, and suppresses aberrant opening of the mitochondrial permeability transition pore (MPTP). By virtue of this mitochondrial repair, the vicious cycle of mutually amplifying oxidative stress and inflammation is successfully disrupted, and microglia are accordingly driven toward the neuroprotective M2 phenotype. In a rat TBI model, the FF‐CPLLA combined with LIPUS treatment significantly alleviated cerebral edema, reduced the extent of cortical damage, increased neuronal survival, and effectively ameliorated neurological deficits and spatial memory impairment. These enhanced therapeutic effects are primarily ascribed to ultrasound‐activated piezoelectric stimulation, although a contributory role of local acoustic‐mechanical coupling effects cannot be excluded. By innovatively applying the mechano‐electrical conversion properties of piezoelectric materials to the regulation of neuroimmune metabolism, this study proposes a therapeutic strategy that targets mitochondrial repair to break the oxidative stress–inflammatory vicious cycle, providing a solid experimental foundation and translational direction for the future development of non‐invasive and precise neural repair therapies.

## 4 Experimental Section

### 4.1 Synthesis of Piezoelectric Fibrous Membranes

#### 4.1.1 Preparation of PLLA Fibrous Membranes

PLLA solid was dissolved in dichloromethane and magnetically stirred at room temperature for 24 h until completely dissolved to obtain a stock solution with a concentration of 100 mg/mL. A 10 mL aliquot of the stock solution was loaded into a plastic syringe connected to a passivated 20‐gauge (inner diameter: 0.58 mm) stainless steel needle, which was then mounted on a metering pump. The electrospinning solution was delivered to the needle tip at a constant rate of 2 mL/h. During electrospinning, the collection distance between the spinneret and the grounded cylindrical collector was set to 14.5 cm, and the collector surface was wrapped with a 5 cm wide conductive aluminum foil. The electric field strength for spinning was maintained at 1.5 kV/cm, supplied by a high‐voltage DC power source, with a voltage of +20 kV applied to the needle tip and −5 kV to the collector end, resulting in a total voltage of 21.75 kV. The rotational speed of the cylindrical collector was set to 100 r/min. After electrospinning for 5 h under ambient conditions, the resulting PLLA fibrous membranes, with a final thickness of approximately 0.02 mm, was vacuum‐dried for 24 h to allow residual organic solvent to evaporate.

#### 4.1.2 Preparation of CPLLA Fibrous Membranes

The synthesized PLLA fibrous membranes sample was placed at the center of the constant temperature zone in a tube furnace. Under a continuous flow of high‐purity nitrogen as a protective atmosphere, the temperature was increased programmatically to 105°C at a constant heating rate of 5°C/min and then maintained at this target temperature for 600 min. After cooling to room temperature, the CPLLA fibrous membranes was obtained.

#### 4.1.3 Preparation of FF‐CPLLA Fibrous Membranes

FmocFF was dissolved in dimethyl sulfoxide (DMSO) to prepare a stock solution with a concentration of 100 mg/mL. Sodium chloride (NaCl) was dissolved in ultrapure water and uniformly dispersed by ultrasonication to obtain a 0.63 mm aqueous solution. The FmocFF/DMSO stock solution was then mixed with the NaCl aqueous solution at a volume ratio of 1:49 and stirred thoroughly to ensure homogeneity. The resulting mixture was applied evenly onto the surface of a flat CPLLA fibrous membranes at a dosage of 80 µL/cm^2^ and allowed to stand at room temperature for 1 h to facilitate hydrogel formation. Finally, the obtained hydrogel was freeze‐dried to yield the FF‐CPLLA composite fibrous membranes, with a final thickness of approximately 0.02 mm.

#### 4.1.4 Characterization of Piezoelectric Fibrous Membranes

Scanning Electron Microscopy (S‐3400N, Hitachi, Japan) was used to characterize the morphology and dimensions of the piezoelectric fibrous membranes. Ultraviolet‐Visible‐Near Infrared Spectrophotometry (UV‐2600, Shimadzu, Japan) was employed to measure the absorption spectra. X‐Ray Diffraction (D8 Advance, Bruker, Germany) was utilized to analyze the crystal structure of the materials. X‐Ray Photoelectron Spectroscopy (ESCALab220i‐XL, VG Scienta, UK) was applied to determine the elemental composition and chemical states. Ultrasonic therapy equipment (T38082, Chattanooga, USA) was used for sonication experiments. Laser scanning confocal microscopy (TCS SP8, Leica, Germany) was employed for fluorescence imaging analysis. Raman spectroscopy (SENTERRA II, Bruker) was utilized to characterize the chemical structure and interactions. Fourier transform infrared spectroscopy (FTIR, Nicolet 6700, Thermo Fisher Scientific) was applied to detect characteristic chemical bonds.

### 4.2 Piezoresponse Force Microscopy (PFM) Measurements

Piezoresponse force microscopy (PFM) measurements were conducted using an MFP‑3D Origin+ atomic force microscope (Oxford Instruments, UK) under ambient conditions. In contact mode, an AC voltage was applied to the conductive probe to acquire PFM amplitude and phase images. Local piezoelectric responses were further characterized by piezoresponse spectroscopy, where a DC bias was superimposed on the AC driving signal to record the amplitude–bias and phase–bias hysteresis loops. The effective piezoelectric coefficient (d_33_) was determined from the linear slope of the amplitude–voltage curve in the central switching region.

### 4.3 Measurement of Open‐Circuit Output Voltage Under Ultrasound

A 1 × 1 cm sample was coated with silver and copper on both sides as electrodes. Copper wires were connected to both electrodes and sealed with polytetrafluoroethylene (PTFE) tape. The fabricated device was exposed to ultrasound at 1 MHz with a 50% duty cycle and varying power intensities (0, 0.1, 0.3, 0.5, 0.7, 1.0, and 1.5 W/cm^2^) using an ultrasonic probe. The resulting piezoelectric voltage was recorded with an oscilloscope.

### 4.4 Measurement of Acoustic Pressure Signals Under Ultrasound

Measurement of acoustic pressure signals under ultrasound was performed using a calibrated needle hydrophone. The hydrophone was positioned at a fixed distance from the ultrasonic probe and immersed in degassed water to record ultrasound‐induced acoustic signals. During the measurements, ultrasound was applied at a frequency of 1.0 MHz with a 50% duty cycle and a fixed acoustic power of 0.3 W for all experimental groups, including the Control, CPLLA, FF, and FF‐CPLLA samples. The time‐domain sound pressure signals were continuously recorded using a digital oscilloscope. To ensure measurement consistency, all experiments were conducted under the same geometric configuration and acoustic conditions. The recorded sound pressure signals were subsequently processed by Fast Fourier Transformation (FFT) to obtain frequency‐domain spectra, and the spectral amplitudes were further integrated over the designated frequency range for quantitative comparison of acoustic output intensity. For acoustic field mapping, the hydrophone was mounted on a 3D motorized translation stage and scanned across the XOY plane at the sample position and the XOZ plane along the ultrasound propagation direction. The pressure distributions in the XOY and XOZ planes were reconstructed, and the mean pressure, maximum pressure, pressure range, and coefficient of variation within the sample ROI were calculated. The −6 dB focal region was defined as the region where the pressure amplitude was ≥50% of the maximum pressure, from which the lateral beam widths, axial focal length, and estimated focal volume were obtained. Possible standing‐wave artefacts were assessed by examining whether the axial pressure profile showed periodic modulation close to λ/2 spacing.

### 4.5 Source and Culture of Rat Microglial Cell Line (HAPI)

The rat microglial cell line HAPI (RRID: CVCL_0F62) was obtained from Shanghai Jinyuan Biotechnology Co., Ltd. (Cat# JY833). Two batches of cells were used in this study, purchased in November 2024 and August 2025, respectively. Both batches tested negative for mycoplasma contamination by routine examination. Cells were cultured in Dulbecco's Modified Eagle Medium (DMEM; Gibco, USA) supplemented with 10% fetal bovine serum (FBS) and 1% penicillin‐streptomycin (Gibco, USA). All cultures were maintained at 37°C in a humidified atmosphere of 5% CO_2_.

### 4.6 Rat Microglial Cell Treatment Groups

Rat microglial cells were seeded in 12‐well plates at a density of 1 × 10^6^ cells per well and incubated for 24 h. The intervention group was treated with 400 µm H_2_O_2_ for 12 h. In the treatment group, rat microglial cells were co‐cultured with piezoelectric thin films and subjected to either ultrasound‐responsive (US) or non‐ultrasound‐responsive conditions. The cells were divided into five groups: I) Control, II) H_2_O_2_, III) H_2_O_2_ + US, IV) H_2_O_2_ + FF‐CPLLA, V) H_2_O_2_ + FF‐CPLLA + US.

### 4.7 CCK‐8 and Live/Dead Cell Staining

Rat microglial cells were seeded into 96‐well plates at a density of 5 × 10^3^ cells per well and incubated for 24 h before receiving the designated interventions. For the CCK‐8 assay, the culture medium was first replaced with 100 µL of fresh medium, after which 10 µL of CCK‐8 solution was added to each well. The plates were then measured for optical density at 450 nm using a Rayto Rt2100c microplate reader. Cell viability and cytotoxicity were separately assessed using a commercial Calcein/PI assay kit (Beyotime, China).

### 4.8 Mitochondrial Membrane Potential (ΔΨm) and MPTP

Rat microglial cells were uniformly seeded on climbing sheets. After 24 h of incubation, corresponding interventions were administered. Changes in mitochondrial membrane potential across different cell groups were detected using JC‐1 (MCE, USA) at the manufacturer's recommended concentration. The opening level of the mitochondrial permeability transition pore (MPTP) was measured in different groups using an MPTP Assay Kit (Beyotime, China).

### 4.9 ATP Levels and MitoSOX Mitochondrial Superoxide

Following the corresponding interventions in rat microglial cells cultured in 12‐well plates, intracellular ATP levels and mitochondrial superoxide were quantified. The measurements were performed using an ATP Assay Kit (Beyotime, China) combined with a chemiluminescence instrument (Thermo Fisher Scientific, USA), and the MitoSOX Red indicator (Thermo Fisher Scientific, USA) was analyzed by flow cytometry, respectively.

### 4.10 MitoTracker Red CMXRos Staining

Rat microglial cells were plated uniformly on confocal dishes (SORFA, China) and subjected to H_2_O_2_ treatment. Subsequently, the cells were stained with a Mito Tracker Red CMXRos (Beyotime, China) by incubation at 37°C for 30 min. After staining, the cells were fixed with 4% paraformaldehyde (PFA) for 15 min and washed three times with phosphate‐buffered saline (PBS). Mitochondrial morphology was examined using a laser scanning confocal microscope (TCS SP8, Leica, Germany).

### 4.11 Cellular ROS Staining

Rat microglial cells were plated on coverslips and treated as described. After 12 h, a Reactive Oxygen Species Assay Kit (Beyotime, China) was used to determine the intracellular ROS levels.

### 4.12 Measurement of MDA and SOD Levels

Rat microglial cells were treated as described above. The malondialdehyde (MDA) content and superoxide dismutase (SOD) activity were subsequently measured using a Lipid Peroxidation MDA Assay Kit (Beyotime, China) and a Total SOD Assay Kit with WST‐8 method (Beyotime, China), respectively, according to the manufacturers’ protocols.

### 4.13 Quantitative Real‐Time PCR Analysis

According to the manufacturer's instructions, total RNA was extracted from the cells of each group employing the Cell Total RNA Isolation Kit (Foregene, USA). Subsequently, cDNA synthesis was performed using the Affinity Script qPCR cDNA Synthesis System (Agilent Technologies, USA). Real‐time quantitative PCR (RT‐qPCR) was conducted in 10 µL reaction volumes containing the synthesized cDNA, forward and reverse primers, and SYBR Green ReadyMix (Thermo Fisher Scientific, USA). The relative expression levels of target RNAs were calculated by the 2^^(−ΔΔCt)^ method, and the primer sequences utilized are listed in Table S3.

### 4.14 Western Blotting

Following lysis of rat microglial cells with RIPA buffer (Beyotime, China) and centrifugation at 13 400 × g (4°C) to obtain total protein, Western blotting was performed as previously described [55]. The membranes were probed with the following primary antibodies at their recommended dilutions: iNOS (1:1000, Proteintech), Arg‐1 (1:5000, Proteintech), NLRP3 (1:1000, CST), Drp1 (1:1000, CST), p‐Drp1^Ser616^ (1:1000, CST), and Mitofusin‐1 (1:1000, CST), Mitofusin‐2 (1:1000, abcam).

### 4.15 Immunofluorescence Staining

After treatment, rat microglial cells were sequentially processed for immunofluorescence staining. Briefly, cells were fixed in warm 4% formaldehyde (15 min) after a PBS wash, followed by permeabilization with ice‐cold methanol (5 min) and blocking with 5% FBS (1 h). Primary antibody incubation was carried out overnight at 4°C using antibodies targeting iNOS (1:500, Proteintech), Arg‐1 (1:500, Proteintech), TOM20 (1:800, Proteintech), p‐Drp1^Ser616^ (1:100, Cell Signaling Technology) and Mitofusin‐2(1:300, abcam). This was followed by a 1‐h incubation in the dark at room temperature with fluorescently conjugated secondary antibodies (1:400). Finally, nuclei were stained with DAPI, and imaging was performed on a confocal laser scanning microscope.

### 4.16 Source of SD Rats

Sprague–Dawley rats (weighing 250–280 g) were purchased from SPF (Beijing) Biotechnology Co., Ltd. After 1 week of acclimatization, the rats were randomly assigned to experimental groups. All animal experiments were reviewed and approved by the Independent Ethics Committee of the Characteristic Medical Center of the Chinese People's Armed Police Force (Ethics Approval No.: 2025‐0006).

### 4.17 Experimental Design and Animal Surgery

Eighty male Sprague–Dawley rats (weighing 250–280 g) were randomly divided into five groups: sham group, TBI group, TBI + US group, TBI + FF‐CPLLA group, and TBI + FF‐CPLLA + US group. All surgical procedures were performed under general anesthesia induced by intraperitoneal injection of 3% sodium pentobarbital. TBI model was established using a CCI device. Subsequently, an enlarged craniotomy was performed above the left parietal window (between the bregma and lambda) using a dental drill. A TBI impactor with a flat metal tip was used to induce moderate traumatic brain injury in rats using the following parameters: impact depth of 2 mm, velocity of 5 m/s, and contact duration of 200 ms. In the piezoelectric fibrous membrane group, after brain injury, a piezoelectric fibrous membrane of appropriate size was applied to cover the injured area. The wound was disinfected with povidone‐iodine and sutured layer by layer. TBI rats were placed on a heating pad until they regained consciousness and then returned to their cages. The sham group underwent only craniectomy. The ultrasound intervention group received treatment at an intensity of 0.3 W/cm^2^, frequency of 1 MHz, duty cycle of 50%, for 2 min per session, once daily (at 9:00 AM).

### 4.18 Brain Tissue Water Content Detection

Rats were anesthetized with 3% sodium pentobarbital. Brains were rapidly extracted, followed by removal of the olfactory bulbs, cerebellum, and lower brainstem. Tissues were immediately weighed to obtain wet weight (WW), then dried in an oven at 110°C for 24 h before reweighing to determine dry weight (DW). Brain water content (%) was calculated using the formula:

Brainwatercontent(%)=[(WW−DW)/WW]×100%

### 4.19 Analysis of Inflammatory Cytokines in Brain Tissue by ELISA

On the third day after surgery, rats were anesthetized using 3% sodium pentobarbital. After cardiac perfusion was performed, the brains were harvested. The brain tissue surrounding the lesion site was dissected and homogenized in sterile PBS supplemented with a protease inhibitor cocktail using an ultrasonic disruptor. The homogenate was centrifuged at 12,000 × g for 10 min at 4°C, and the resulting supernatant was collected. The concentrations of the inflammatory cytokines IL‐1β, IL‐6, and TNF‐α were subsequently measured in the supernatant using appropriate commercial rat ELISA kits (Beyotime, China) by strictly following the manufacturer's guidelines.

### 4.20 Fluorescent Staining of Brain Tissue Sections

Deparaffinized brain sections were subjected to antigen retrieval by heating in citrate buffer. After being blocked with normal goat serum, the sections were incubated overnight at 4°C with primary antibodies targeting iNOS (1:500, Proteintech), CD206 (1:500, Proteintech), and IBA‐1 (1:100, Abcam). This was followed by a 1‐h incubation at room temperature with a fluorescent secondary antibody (1:400, Proteintech). Nuclei were counterstained with DAPI, and following three PBS washes, the sections were mounted using an anti‐fade medium. Visualization and image acquisition were carried out with a Leica microscope and its associated software.

### 4.21 Transmission Electron Microscopy of Brain Tissue

At 3 days post‐operation, rats were anesthetized using 3% sodium pentobarbital and subjected to intracardiac perfusion fixation with a solution containing 4% paraformaldehyde and 2.5% glutaraldehyde. Cortical tissue samples (≈1 mm^3^) from the lesion periphery were then dissected and immersion‐fixed overnight at 4°C in 2.5% glutaraldehyde. Following primary fixation, the samples underwent post‐fixation in 1% osmium tetroxide for 2 h at 4°C. The tissues were then dehydrated through a graded ethanol series and embedded in Epon 812 resin. Ultrathin sections (60–80 nm) were prepared, stained with uranyl acetate and lead citrate, and ultimately examined under a Hitachi HT7700 transmission electron microscope for image acquisition.

### 4.22 Modified Neurological Severity Score (mNSS)

Neurological function was assessed in rats using the mNSS [56] on days 3, 7, 14, and 28 after surgery. The mNSS is a composite scoring system that encompasses motor, sensory, reflex, and balance tests, with well‐defined criteria.

### 4.23 Morris Water Maze Experiment

Apparatus and Training: The Morris water maze consisted of a circular pool (diameter: 1.5 m) filled with opaque, warm water deep enough to conceal a platform. Over five consecutive days, each rat completed four trials per day. Each trial provided a maximum of 90 s to locate the hidden platform after being placed in the water from a random quadrant. The rat remained on the platform for 15 s after finding it or being guided to it. Escape latency and swimming path were recorded. Probe Trial: On the sixth day, a probe trial was conducted with the platform removed. Each rat swam freely for 90 s, during which the time spent in the target quadrant, the number of crossings over the platform's original location, and the average swimming speed were recorded.

### 4.24 H&E Staining and Nissl Staining

Paraffin‐embedded tissue sections were deparaffinized and subsequently stained using Hematoxylin and Eosin (H&E) Staining Kit and Nissl Staining Kit (both from Beyotime, China), strictly following the manufacturers’ protocols. The stained sections were evaluated for general histopathological morphology and changes in neuronal density.

### 4.25 RNA Sequencing and Analysis

In animal experiments, peri‐injury cortical tissue was collected from rats in the Sham, TBI, and TBI + FF‑CPLLA + US groups at postoperative day 3 (*n* = 5 per group). All samples were snap‐frozen in liquid nitrogen and stored at −80°C until RNA extraction. Total RNA was extracted using TRIzol reagent. RNA integrity was assessed using an Agilent 2100 Bioanalyzer. Sequencing libraries were prepared and sequenced on an Illumina NovaSeq 6000 platform. Raw reads were filtered using fastp (v0.19.7) with default parameters to remove adapter sequences, reads containing N bases, and low‐quality reads (Qphred ≤ 5 over more than 50% of read length). Clean reads were aligned to the rat reference genome (Ensembl 102, Rattus_norvegicus_Rnor_6_0) using HISAT2 (v2.2.1) with default parameters. Gene‐level read counts were quantified using featureCounts (v2.0.6), and FPKM values were calculated for data visualization. Differential expression analysis was performed using the DESeq2 package (v1.42.0), with |log_2_(fold change)| ≥ 1 and adjusted *p* < 0.05 set as the thresholds for statistical significance. Principal component analysis (PCA), hierarchical clustering heatmaps, volcano plots, and Venn diagrams were generated to visualize transcriptomic profiles. Gene Ontology (GO) and Kyoto Encyclopedia of Genes and Genomes (KEGG) enrichment analyses were conducted using clusterProfiler (v4.8.1) to identify significantly enriched biological processes and signaling pathways.

### 4.26 Statistical Analysis

We performed all statistical analyses with GraphPad Prism software (version 9.0). Continuous data are presented as mean ± SD. For comparisons among groups, one‐way ANOVA followed by Duncan's test was applied. Statistical significance was set at *p* < 0.05, indicated as *p < 0.05, **p < 0.01, ***p < 0.001, and ****p < 0.0001.

## Conflicts of Interest

The authors declare no conflicts of interest.

## Supporting information




**Supporting File**: advs76756‐sup‐0001‐SuppMat.docx.

## Data Availability

The data that support the findings of this study are available from the corresponding author upon reasonable request.
