# Supplementary material for: Ultrasound‐Responsive Piezoelectric Fibrous Membrane Promotes Neurological Recovery After TBI by Modulating Microglial Polarization via Restoring Mitochondrial Dynamic Homeostasis
Source: Adv Sci (Weinh). 2026 Sep 21;13(58):e00081. doi: 10.1002/advs.202600081 (PMC13591349; doi:10.1002/advs.202600081)
Supplement: Supplementary file 1 [file ADVS-13-e00081-s001.docx]

Supporting Information ：

Ultrasound-Responsive Piezoelectric Fibrous Membrane Promotes Neurological Recovery after TBI by Modulating Microglial Polarization via Restoring Mitochondrial Dynamic Homeostasis

*WeiLi^#^, Runzhe Huang^#^, Qingyuan Wu^#^, Pengbo Zhou, Dangli Ren,Hanjie Niu, Jingjing Wang, Zemeng Li, Hongtao Sun*, Huiyu Liu**

Figure S1: Interfiber spacing (A) and porosity (B) of CPLLA and FF-CPLLA fibrous membranes. Data are presented as mean ± SD; n = 5.

Figure S2: XPS core-level spectra: (A) FF O 1s, (B) CPLLA O 1s, (C) FF-CPLLA O 1s, (D) FF N 1s, (E) CPLLA N 1s, (F) FF-CPLLA N 1s.

Figure S3: Elemental mapping of C, N, O: (A) FF hydrogel, (B) FF-CPLLA fibrous membranes.

Figure S4: SEM images of FF-CPLLA fibrous membranes after degradation in simulated cerebrospinal fluid (CSF) for (A) 1 week, (B) 2 weeks, (C) 3 weeks, and (D) 4 weeks.

Figure S5: In vitro degradation of FF-CPLLA fibrous membranes in simulated CSF: (A) Time-dependent mass loss of the membranes during 4 weeks of incubation, (B) Corresponding lactic acid concentration released into the supernatant. Data are presented as mean ± SD; n = 3.

Figure S6: Stress-strain curves of CPLLA and FF-CPLLA.

Figure S7: Force-displacement curves of CPLLA and FF-CPLLA.

Figure S8: Young’s modulus of CPLLA and FF-CPLLA. Data are presented as mean ± SD; n = 3.

Figure S9: XRD pattern of CPLLA.

Figure S10: Piezoresponse force microscopy (PFM) characterization: (A) AFM image of PLLA fibrous membranes; Scale bar, 2 μm. (B) PFM amplitude image of PLLA fibrous membranes; Scale bar, 2 μm. (C) PFM phase image of PLLA fibrous membranes; Scale bar, 2 μm. (D) AFM image of CPLLA fibrous membranes; Scale bar, 2 μm. (E) PFM amplitude image of CPLLA fibrous membranes; Scale bar, 2 μm. (F) PFM phase image of CPLLA fibrous membranes; Scale bar, 2 μm. (G) AFM image of FF hydrogel; Scale bar, 1 μm. (H) PFM amplitude image of FF hydrogel; Scale bar, 1 μm. (I) PFM phase image of FF hydrogel. Scale bar, 1 μm.

Figure S11: Piezoelectric properties: (A) Amplitude curve and piezoelectric response phase curve of the PLLA fibrous membranes; (B) Amplitude curve and piezoelectric response phase curve of the CPLLA fibrous membranes; (C) Amplitude curve and piezoelectric response phase curve of the FF hydrogel.

Figure S12: The FFT spectra of the signals corresponding to the Control group, FF hydrogel, CPLLA fibrous membranes, and FF-CPLLA fibrous membranes.

Figure S13: Photograph of the experimental setup for measuring the voltage output generated by FF-CPLLA fibrous membranes under transcranial ultrasound stimulation via an *ex vivo* skull.

Figure S14: Voltage output of FF-CPLLA fibrous membranes in different media. (A) Representative voltage-time curves of FF-CPLLA in air, CSF, and an ex vivo skull upon ultrasound stimulation. (B) Corresponding statistical comparison of the voltage outputs. Data are presented as mean ± SD; n = 5.

Figure S15: *In vivo* and *in vitro* temperature monitoring during ultrasound stimulation. (A)IR thermal images at different time points; (B) Real-time temperature profiles recorded in vivo and in vitro. Data are presented as mean ± SD; n = 3.

Figure S16. Two-dimensional acoustic-field maps and linear pressure profiles at the experimental sample location. (A) Transverse X-Y map of peak acoustic pressure; (B) Axial X-Z map of peak acoustic pressure; (C) Peak-pressure profile extracted through the focal region along the transverse Y direction; (D) Peak-pressure profile extracted through the focal region along the axial Z direction.

Figure S17. Cytotoxicity evaluation of different materials co-cultured with rat microglial cells for 24, 48, and 72 hours via the CCK-8 assay (n=5). Quantitative data are presented as mean ± SD.

Figure S18. Evaluation of the cytotoxicity of different materials on rat microglial cells by live/dead cell staining. (A) Representative fluorescence images stained with Calcein-AM (green, live cells) and propidium iodide (PI, red, dead cells). Scale bar, 50 μm. (B) Quantitative cell viability of rat microglial cells after co-culture with PLLA, CPLLA, and FF-CPLLA (mean ± SD, n=3).

Figure S19. Cell viability of rat microglial cells measured by the CCK-8 assay at 12 hours after intervention with different ultrasound intensities (n=4). Quantitative data are presented as mean±SD.

Figure S20. Effect of different ultrasound intensities on rat microglial cells observed under an optical microscope.

Figure S21. Effects of different concentrations of H_2_O_2_ on the viability of rat microglial cells (n=4). Quantitative data are presented as mean ± SD.

Figure S22. Assessment of mitochondrial membrane potential in rat microglial cells after intervention with 400 μM H_2_O_2_ for different durations by flow cytometry with JC-1 staining. (A) Alterations in ΔΨm of rat microglial cells stimulated by H_2_O_2_ for 1, 3, 6, 9, and 12 hours.

Figure S23.Whole-transcriptome analysis of peri-injury cortical tissue after TBI and piezoelectric treatment. (A) Principal component analysis (PCA) of RNA-seq data from the Sham, TBI, and Tre groups (n=5 per group). (B) Hierarchical clustering heatmap of differentially expressed genes (DEGs) across the three groups. (C) Venn diagram illustrating the overlap of DEGs among the three groups. (D–E) Volcano plots of DEGs for the pairwise comparisons of (D) TBI vs. Sham and (E) TBI vs. Tre.

Figure S24. Gene Ontology (GO) enrichment analysis of the 126 DEGs identified from the TBI vs. Tre comparison.

Figure S25. Figure S24. KEGG pathway enrichment analysis of the 126 DEGs from the TBI vs. Tre comparison.

Figure S26. Validation of NLRP3 inflammasome protein expression. (A) Representative Western blot bands of NLRP3 in the Sham, TBI, and Tre groups at postoperative day 3. (B) Quantification of NLRP3 protein expression . Data are presented as mean ± SD , n = 3.

Figure S27. Physical Status and Tissue Response of the Implanted FF-CPLLA Fibrous Membrane at Different Time Points Post-Surgery. At all observed time points, the FF-CPLLA fibrous membrane remained intact, covering the lesion site without displacement. No adhesive encapsulation or significant inflammatory proliferative membrane formed between the implant and the surrounding brain tissue. Over time, the membrane progressively integrated tightly with the adjacent skull and host tissue, ultimately forming a sealed, dura mater-like covering over the injured cortical surface.

Figure S28. Representative H&E staining of normal brain tissue sections after 28‑day implantation of the FF‑CPLLA membrane with or without ultrasound stimulation.

Figure S29. Degradation Profile of the Implanted FF-CPLLA Fibrous Membrane in a Brain Injury Model. Throughout the study, the fibrous membrane remained macroscopically intact without structural disintegration. At postoperative day 7, it maintained structural integrity and could be peeled off relatively completely. By day 14, the membrane became tightly integrated with the surrounding tissue, exhibiting an initial reduction in toughness. At day 28, significantly increased brittleness was observed, making it prone to fragmentation during retrieval and difficult to harvest intact.

**Table S1.** Quantitative acoustic parameters within the sample region of interest under the experimental ultrasound condition.

| **Parameter** | **Symbol** | **Report** |
| --- | --- | --- |
| Maximum peak pressure at the sample plane | P_peak,max_ | 35.6 kPa |
| Mean peak pressure within the sample ROI | P_peak,ROI mean_ | 19.4 ± 6.4 kPa |
| Peak pressure range within the sample ROI | P_peak,ROI range_ | 10.9-35.6 kPa |
| Pressure coefficient of variation within the sample ROI | CV | 0.33 |
| Maximum RMS pressure at the focus | P_rms,max_ | 25.2 kPa |
| RMS pressure within the sample ROI | P_rms,ROI_ | 13.7 ± 4.5 kPa |
| −6 dB lateral beam widths | W_x_,W_y_(P≥0.5P_max_) | 15.4×15.1 mm |
| −6 dB axial focal length | L_z_(P≥0.5P_max_) | 9.3 mm |
| Estimated −6 dB focal volume | V_−6dB_ | 1132.3 mm^3^ |
| Acoustic wavelength in water | λ | 1.48 mm |
| Expected standing-wave node spacing | λ/2 | 0.74 mm |
| Standing-wave assessment |  | No regular λ/2-spaced axial modulation |

**Table S2.** Blood biochemical parameters of rats in different groups after 4 weeks.

| Blood biochemical parameters | Control | FF-CPLLA | FF-CPLLA + US |
| --- | --- | --- | --- |
| ALT (U/L) | 49.67 ± 2.60 | 49.30 ± 2.29 | 49.38 ± 4.06 |
| AST (U/L) | 149.68 ± 1.95 | 149.88 ± 2.63 | 149.82 ± 3.29 |
| LDH (U/L) | 850.86 ± 5.88 | 845.48 ± 8.07 | 845.93 ± 8.98 |
| TBIL (μmol/L) | 0.63 ± 0.07 | 0.60 ± 0.57 | 0.63 ± 0.11 |
| TC (mmol/L) | 1.50 ± 0.15 | 1.46 ± 0.09 | 1.52 ± 0.09 |
| CREA (μmol/L) | 28.93 ± 1.22 | 28.06 ± 1.04 | 27.78 ± 1.09 |
| UREA (mmol/L) | 5.37 ± 0.23 | 5.28 ± 0.26 | 5.26 ± 0.22 |
| UA (μmol/L) | 48.25 ± 0.95 | 48.19 ± 1.43 | 45.60 ± 2.46 |

**Table S3.** Primer sequences for RT-qPCR.

| Gene |  | Primer sequence |
| --- | --- | --- |
| IL-1β | Forward | 5’-ATGATGGCTTATTACAGTGGCAA-3’ |
|  | Reverse | 5’-GTCGGAGATTCGTAGCTGGA-3’ |
| IL-6 | Forward | 5’-GGCCCTTGCTTTCTCTTCG-3’ |
|  | Reverse | 5’-ATAATAAAGTTTTGATTATGT-3’ |
| TNF-α | Forward | 5’-CCTCTCTCTAATCAGCCCTCTG-3’ |
|  | Reverse | 5’-GAGGACCTGGGAGTAGATGAG-3’ |
| β-actin | Forward | 5’-TTGATCTTCATTGTGCTGGGTG-3’ |
|  | Reverse | 5’-CTTCCTGGGCATGGAATCCT-3’ |
